# Pathogen Specific, IRF3-Dependent Signaling and Innate Resistance to Human Kidney Infection

**DOI:** 10.1371/journal.ppat.1001109

**Published:** 2010-09-23

**Authors:** Hans Fischer, Nataliya Lutay, Bryndís Ragnarsdóttir, Manisha Yadav, Klas Jönsson, Alexander Urbano, Ahmed Al Hadad, Sebastian Rämisch, Petter Storm, Ulrich Dobrindt, Ellaine Salvador, Diana Karpman, Ulf Jodal, Catharina Svanborg

**Affiliations:** 1 Department of Microbiology, Immunology and Glycobiology, Institute of Laboratory Medicine, Lund University, Lund, Sweden; 2 Singapore Immunology Network (SIgN), Biomedical Sciences Institutes, Agency for Science, Technology, and Research (A*STAR), Immunos, BIOPOLIS, Singapore, Singapore; 3 Institute for Molecular Biology of Infectious Diseases, Julius-Maximilians-University Würzburg, Würzburg, Germany; 4 Department of Pediatrics, Clinical Sciences Lund, Lund University, and Lund University Hospital, Lund, Sweden; 5 Pediatric-Uronephrology Center, Queen Silvia Children's Hospital, University of Gothenburg, Sweden; Massachusetts General Hospital and Harvard Medical School, United States of America

## Abstract

The mucosal immune system identifies and fights invading pathogens, while allowing non-pathogenic organisms to persist. Mechanisms of pathogen/non-pathogen discrimination are poorly understood, as is the contribution of human genetic variation in disease susceptibility. We describe here a new, IRF3-dependent signaling pathway that is critical for distinguishing pathogens from normal flora at the mucosal barrier. Following uropathogenic *E. coli* infection, *Irf3^−/−^* mice showed a pathogen-specific increase in acute mortality, bacterial burden, abscess formation and renal damage compared to wild type mice. TLR4 signaling was initiated after ceramide release from glycosphingolipid receptors, through TRAM, CREB, Fos and Jun phosphorylation and p38 MAPK-dependent mechanisms, resulting in nuclear translocation of IRF3 and activation of IRF3/IFNβ-dependent antibacterial effector mechanisms. This TLR4/IRF3 pathway of pathogen discrimination was activated by ceramide and by P-fimbriated *E. coli*, which use ceramide-anchored glycosphingolipid receptors. Relevance of this pathway for human disease was supported by polymorphic *IRF3* promoter sequences, differing between children with severe, symptomatic kidney infection and children who were asymptomatic bacterial carriers. *IRF3* promoter activity was reduced by the disease-associated genotype, consistent with the pathology in *Irf3^−/−^* mice. Host susceptibility to common infections like UTI may thus be strongly influenced by single gene modifications affecting the innate immune response.

## Author Summary

The host immune system must identify pathogens and defeat them through TLR-dependent signaling pathway activation, while distinguishing them from commensal flora. Contrary to current dogma, the host cannot solely use “pattern recognition” since the microbial molecules involved in such recognition are present on pathogens and commensals alike. We identify here a pathogen-specific mechanism of TLR4 activation and signaling intermediates in this pathway, leading to IRF3-dependent transcription of innate immune response genes. We show in knockout mice that *Irf3* deficiency causes severe tissue pathology and that effector functions controlled by IFNβ are involved. Finally, in highly disease-prone pyelonephritis patients we found a high frequency of *IRF3* promoter polymorphism compared to asymptomatic bacterial carriers or controls. The polymorphisms influenced promoter activity in reporter assays, suggesting that they are functionally important. Urinary tract infections are among the most common bacterial infections in man, and are a major cause of morbidity and mortality. A subset of disease-prone individuals is at risk for recurrent disease, severe renal dysfunction and end-stage renal disease. At present, there is no method to identify disease-prone infants and to prevent future morbidity and renal damage. The genetic and functional studies described here indicate that genetic variation in *IRF3* influences individual susceptibility to kidney infection and might serve as a new tool for future risk assessment in this patient group.

## Introduction

Despite significant advances in the understanding of genetic variation, common infections are often regarded as too complex for genetic analysis. While single gene defects have a major impact on host susceptibility to classic infections like malaria [1], the extent to which susceptibility to diarrhea, respiratory tract and urinary tract infection (UTI) is genetically controlled remains unclear. Critical to the understanding of host resistance and genetic control is the mucosal route of these infections and the molecular interactions through which mucosal tissues are perturbed. UTI serve as a particularly useful model to identify genetic variants contributing to host susceptibility, as innate immunity controls the antimicrobial defense and molecular mechanisms of host parasite interaction are understood in great detail [2], [3]. The disease response to uropathogenic *Escherichia coli* is initiated through fimbriae-mediated adherence, and the expression of P fimbriae distinguishes the pathogenic strains from non-virulent bacteria, which colonize the same mucosal sites.

TLRs control the survival of complex organisms by balancing protective against destructive forces of innate immunity. During infection, each TLR recognizes a relatively small number of ligands, including conserved microbial patterns (PAMPs) [4]. The horseshoe-shaped, leucine-rich, extracellular TLR domain and its co-receptors are involved in recognition of proteins, as well as lipids, carbohydrates and nucleic acids [5], [6], [7]. At mucosal sites, where the bulk of microbial challenge occurs, PAMP recognition is non-functional, however, and does not explain how mucosal TLRs distinguish pathogenic microbes from members of the normal flora [8]. Pathogen-specific TLR responses to mucosal pathogens require receptors that exclusively engage virulence ligands and signaling pathways that activate a pathogen-specific defense [8]. For example, uropathogenic *E. coli* adhere to mucosa via glycosphingolipid receptors for P fimbriae, thereby activating a TLR4-dependent but LPS/CD14-independent innate immune response in epithelial cells [9].

Signaling through cell surface sphingolipids involves ceramide, the membrane anchor and a ubiquitous component of cell membranes [10], [11]. The generation of ceramide within rafts alters their biophysical properties and results in the formation of large ceramide-enriched membrane platforms, clustering receptor molecules and facilitating signal transduction following receptor stimulation [12]. Endogenous SMases, activated by many infectious agents, cleave ceramide from the extracellular choline-rich domain of sphingomyelin [13], [14], [15], [16], [17] and activate the “ceramide-signaling pathway”, which is conserved from yeast to humans [18]. In addition, pathogens that utilize the extracellular domain of glycosphingolipids as receptors may release ceramide after bacterial binding, as first described for P-fimbriated, uropathogenic *E. coli* [9], [17], [19]. Ceramide activates a TLR4-dependent innate immune response [17], similar to infection-mediated activation, and we have proposed that ceramide acts as a signaling intermediate between the pathogen-specific receptors and TLR4 [9], [17], [19]. The molecular mechanisms in this important signal transduction need to be identified, however.

UTIs affect >150 million adults each year and about 5% of children <12 years of age. Severe kidney infections like acute pyelonephritis (APN) are accompanied by life-threatening urosepsis in about 30% of adults. Children may develop renal scars, which are associated with long-term morbidity including hypertension, complications of pregnancy, and renal failure if scarring is extensive. Despite the urgent need, no tools exist at present to identify children at risk of developing recurrent acute pyelonephritis and renal scarring. Host resistance to UTI is controlled by the innate immune system, through Toll-like receptor (TLR) activation [8], [9], [20]. Previous studies have shown that TLR4-deficient mice develop asymptomatic carriage rather than severe disease [8], [20], [21], suggesting that disturbances in TLR4 signaling may alter the innate immune dependent host defense [22].

This study examined TLR4 activation by ceramide and P fimbriated *E. coli* and characterized this signaling pathway. We propose that ceramide interacts directly with TLR4, activates TRAM phosphorylation followed by nuclear translocation of IRF3. Furthermore, we show that IRF3 dependent innate immunity is essential for the host defense, as *Irf3* knockout mice develop severe kidney infection. Finally, we show that *IRF3* promoter polymorphisms are more common in APN prone patients than in those who become asymptomatic bacterial carriers. We propose that this pathway offers a model of how TLR4 may distinguish pathogens from commensals at the mucosal level, through modification of pathogen recognition receptors, adaptors and transcription factors.

## Results

### Increased susceptibility to acute pyelonephritis in *Irf3^−/−^* mice

**Figure 1:**
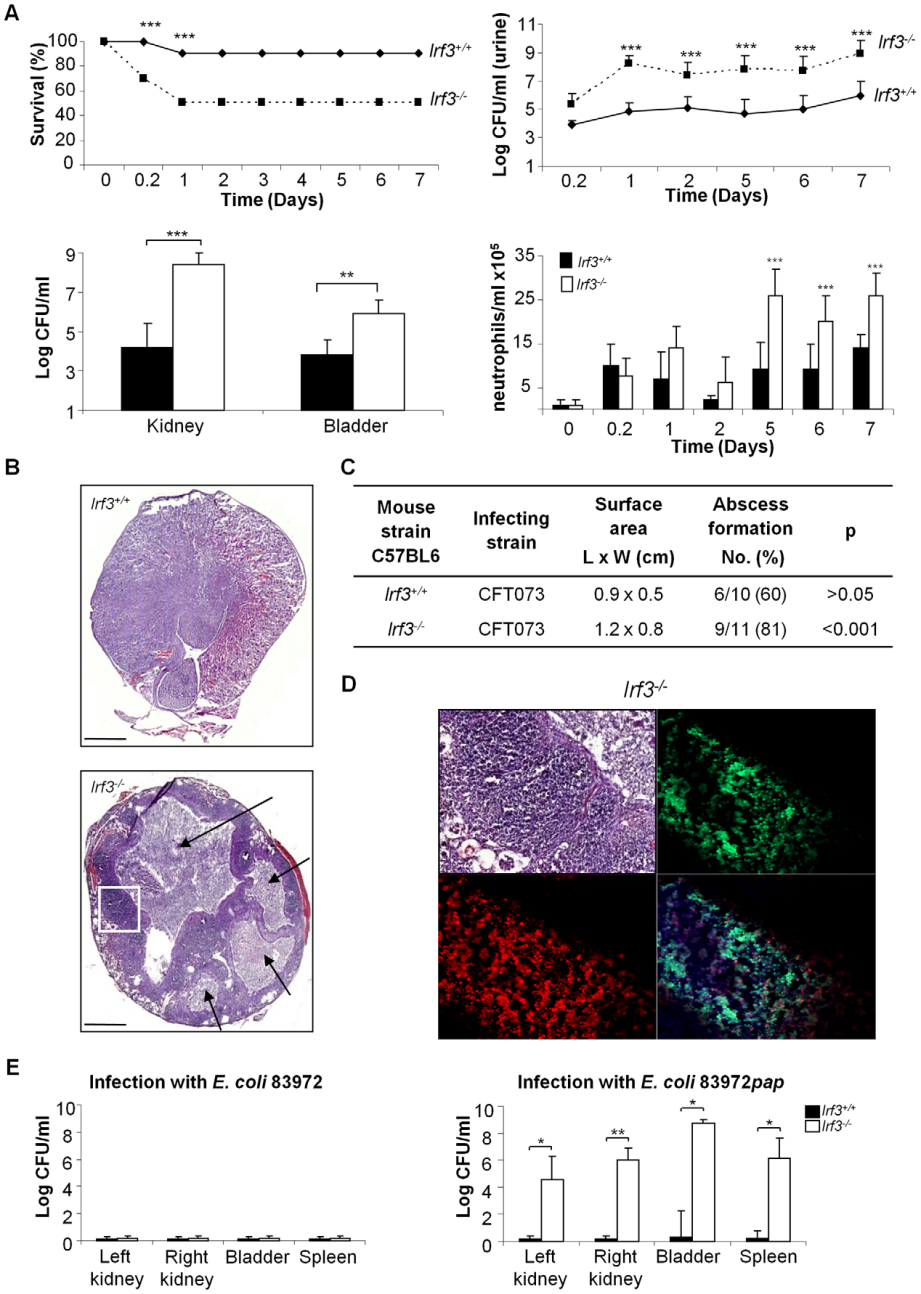
Increased mortality and tissue damage in *Irf3^−/−^* mice after experimental UTI. Uropathogenic *E. coli* CFT073 established acute pyelonephritis more efficiently in *Irf3^−/−^* (n = 11) compared to *Irf3^+/+^* (n = 10) mice. Panel A shows increased mortality (*** p<0.001), bacterial numbers in urine, kidneys and bladders (** p<0.01, *** p<0.001) and neutrophil influx (*** p<0.001). Geometric means ± SEMs. Panels B–D show destruction of renal tissue in *Irf3^−/−^* mice. Sections were stained with hematoxylin-eosin or specific antibodies (neutrophils = green, NIMP-R14, PapG adhesin = red, synthetic PapG peptide antibodies and Nuclear Hoechst staining = blue). Scale bar = 200 mm. Abscesses are indicated by arrows. Panel E shows that non-virulent *E. coli* 83972 are cleared with similar efficiency by wt (n = 8) and *Irf3^−/−^* (n = 8) mice. In contrast, a P-fimbriated transformant *E. coli* 83972*pap*, triggers IRF3-dependent disease. Geometric means ± SEMs (** p<0.01).

In a genetic screen of innate immune effector genes downstream of TLR4, we identified IRF3 as a major determinant of host susceptibility. *Irf3^−/−^* and *Irf3^+/+^* mice were infected via the urinary tract mucosal route with the uropathogenic *E. coli* strain CFT073 [23]. The *Irf3^−/−^* mice developed more severe disease than wild type (wt) *Irf3^+/+^* mice. Acute mortality was higher (50% after 24 hours) and bacterial clearance was significantly impaired, with higher bacterial counts in urine, kidneys and bladders (Figure 1A, p<0.001). Abscess formation was also more extensive in *Irf3^−/−^* than in wt mice (day 7 post-infection, Figure 1B–C, p<0.001). In *Irf3^−/−^* mice, abscesses were diffuse, destroying large tissue areas while in wt mice abscesses were morphologically distinct from surrounding healthy tissue (Figure 1B).

Renal abscess formation is caused, in part, by an imbalance between neutrophil recruitment and exit from the tissues [24], [25]. The kinetics of early neutrophil recruitment did not differ between wild type and *Irf3^−/−^* mice (Figure 1A), but later, neutrophil recruitment subsided in wt mice but remained elevated in *Irf3^−/−^* mice. In tissue sections from *Irf3^−/−^* mice, neutrophils were detected throughout the abscesses and P-fimbriated bacteria were interspersed among the neutrophils, as shown by PapG adhesin-specific antibody detection (Figure 1D, for negative control, see Figure S1). Wt mice, in contrast, had discrete, neutrophil aggregates with fewer bacteria. The results suggest that Irf3 is essential for a functioning innate immune defense against UTI, to maintain tissue integrity and to clear mucosal *E. coli* infection.

To examine if the IRF3-dependent immune response discriminates uropathogenic *E. coli* from non-pathogenic bacteria, we inoculated wt and *Irf3^−/−^* mice with the prototypical asymptomatic bacteriuria strain *E. coli* 83972, which lacks functional UTI-associated virulence factors, including P fimbriae [26], [27], [28], [29]. Both wt and *Irf3^−/−^* mice cleared infection rapidly, with no difference in bacterial counts (Figure 1E) and no significant neutrophil recruitment (data not shown). As P fimbriae are essential virulence factors, present in up to 100% of *E. coli* strains causing urosepsis [30], [31], we subsequently examined if P fimbriae activate the IRF3 pathway. The asymptomatic carrier strain *E. coli* 83972 was transformed with a chromosomal copy of the *pap* gene cluster. We compared disease severity and bacterial counts between wt and *Irf3^−/−^* mice infected with *E. coli* 83972*pap*. The *Irf3^−/−^* mice developed acute, symptomatic disease with sepsis and had dramatically increased bacterial numbers in bladders, kidneys and spleens (Figure 1E, p<0.05), compared to wt mice, which were resistant to infection with *E. coli* 83972*pap*.

The results show that the IRF3-dependent response distinguishes pathogenic *E. coli* from non-pathogenic strains and suggest that the expression of a single virulence factor like P fimbriae enables the host to recognize a potential pathogen and to activate this response.

### Ceramide/TLR4 interactions and TRAM phosphorylation

**Figure 2:**
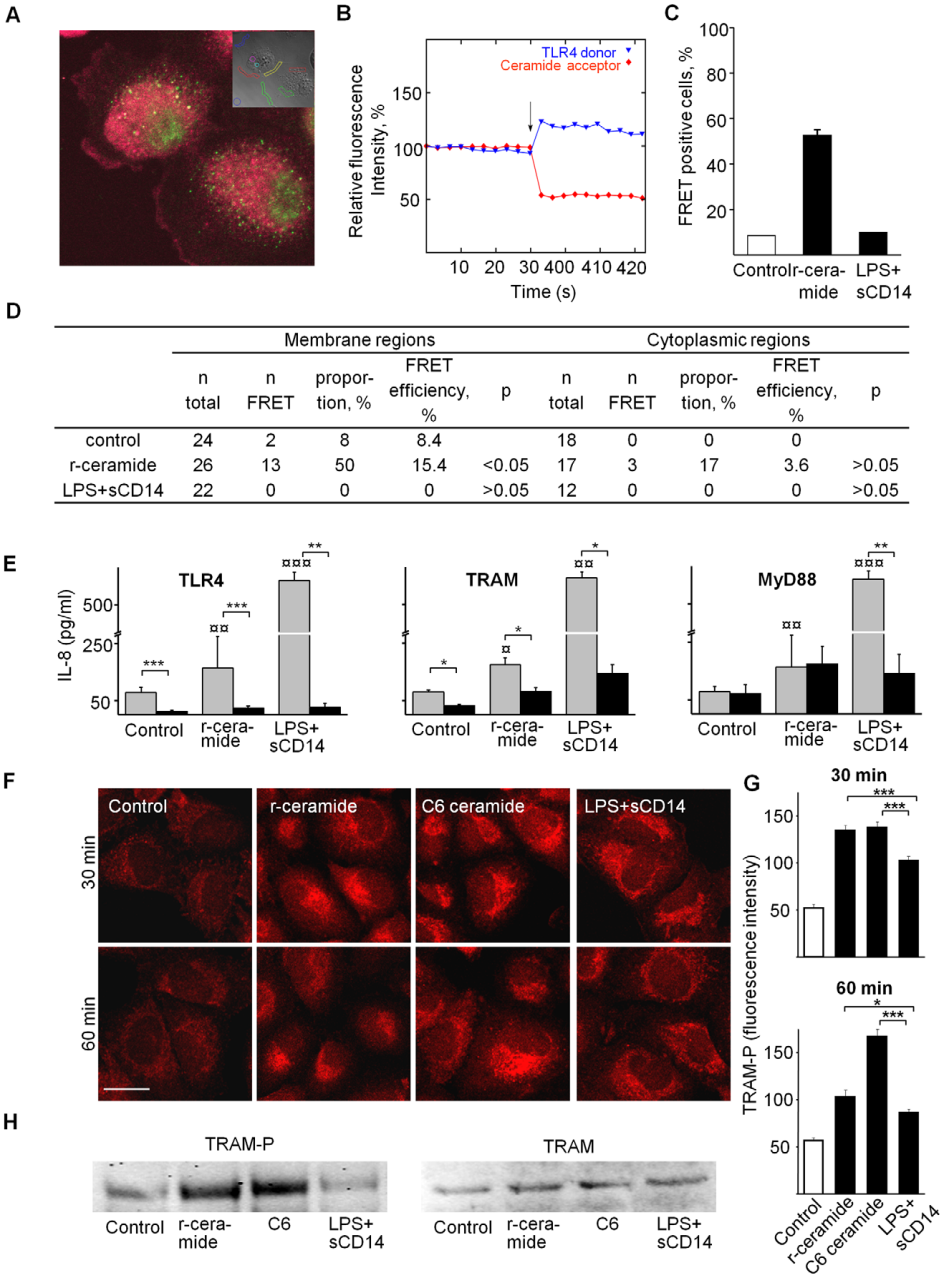
Ceramide-TLR4 interactions, adaptors and TRAM phosphorylation. TLR4 and native ceramide were labeled with specific primary antibodies, followed by Alexa fluor-488 (donor) and Alexa fluor-568 (acceptor)-labeled secondary antibodies, respectively. Acceptor bleaching was quantified by confocal microscopy, comparing target membrane regions (inset) and cytoplasmic control areas. Panel A shows membrane and cytoplasmic staining for TLR4 (green) and ceramide (red). Panel B shows recording of donor and acceptor channels, ten times before and after acceptor bleaching (arrow). The intensity at time 0 was set to 100%. Panel C compared cells exposed to LPS+sCD14 (10+1µg/ml) or r-ceramide (SMase, 1 U/ml, 1 h). Panel D shows an increase in membrane FRET signals after ceramide release, but not after LPS+sCD14 treatment. Panel E shows siRNA silencing (*black*, 72 h transfection) of TLR4, TRAM or MyD88 and resulting inhibition of IL-8 responses in A549 cells, stimulated with SMase (1 U/ml) or LPS+sCD14 (10+1µg/ml). Cytokine responses (Medians±SEMs ≥3 experiments) were compared to irrelevant siRNA-transfected cells (*grey*, * p<0.05, **p<0.01, ***p<0.001) which gave significant IL-8 response compared to background level (¤p<0.05, ¤¤p<0.01, ¤¤¤p<0.001). Panel F shows TRAM phosphorylation (TRAM-P) after r-ceramide, C6 ceramide or LPS+sCD14 exposure (primary polyclonal rabbit-TRAM-P antibodies and secondary anti-rabbit-Alexa fluor-568-labeled antibodies), for a broader view see Figure S3. Panel G shows TRAM-P fluorescence intensity quantified with LSM 510 software (mean±SEM, n = 30 cells/treatment, * p<0.05, *** p<0.001). (H) TRAM-P expression in A549 cells stimulated for 45 min with the indicated agonists compared to total TRAM levels (Western blot).

P fimbriae bind to glycosphingolipid receptors and trigger ceramide release [9]. To investigate the mechanism of pathogen-specific TLR4/IRF3 signaling activation, we examined if ceramide and TLR4 interact after ceramide release from membrane glycosphingolipids. We treated A498 kidney epithelial cells with sphingomyelinase (SMase) for one hour, to release ceramide (r-ceramide) from the extracellular phosphocholine domain of sphingomyelin [32] (Figure 2A–D). We labeled TLR4 and native ceramide with specific primary antibodies followed by Alexa fluor-488 (donor) and Alexa fluor-568 (acceptor)-labeled secondary antibodies, respectively. In unstimulated cells (no SMase treatment), where ceramide remains bound to sphingomyelin, we detected no FRET signal. After SMase treatment, we recorded a significant FRET signal (Figure 2A–D, 50% FRET-positive cells compared to 8% for unstimulated cells, p<0.05), with most of the FRET-positive regions localized in the plasma membrane. LPS and soluble CD14 (sCD14) stimulation, in contrast, did not stimulate a FRET signal above background (p>0.05, compared to unstimulated cells). sCD14 was used, as the uroepithelial cells lack membrane-bound CD14 and respond poorly to LPS [33]. These results suggest that ceramide interacts with TLR4 after release from membrane glycosphingolipids.

To examine the ceramide-induced TLR4 signaling pathway, we used RNA interference to suppress specific genes (Figure 2E, siRNA used for transfection are listed in Table S1 in Supporting Information S1; for knockdown efficiency compared to control cells transfected with irrelevant siRNA, see Figure S2). First, suppression of TLR4 expression abrogated the innate immune response to r-ceramide (p<0.001), confirming that this pathway is TLR4 dependent. Secondly, TRAM siRNA inhibited the responses to r-ceramide (p<0.05 compared to the siRNA control). MyD88-specific siRNA did not alter the ceramide response (p>0.05 compared to the siRNA control) but did reduce the response to LPS+sCD14 (p<0.05), as did TLR4- and TRAM-specific siRNAs.

To further investigate ceramide-induced TLR4 signaling, TRAM phosphorylation (TRAM-P) was quantified by confocal microscopy, using polyclonal phospho-specific anti-TRAM antibodies (Figure 2F–G and Figure S3). We detected an increase in TRAM-P staining in cells exposed to r-ceramide or exogenous, water-soluble C6 ceramide; staining had a granular appearance and was most intense in the perinuclear area. By Western blot analysis (Figure 2H), a band corresponding to TRAM-P was increased in cells exposed to C6 and r-ceramide compared to unstimulated cells but total TRAM levels were not altered. LPS+sCD14 triggered weaker TRAM phosphorylation, as shown by confocal microscopy (p<0.001 compared to r-ceramide) and by Western blot. The results indicate that ceramide triggers TRAM phosphorylation more efficiently than LPS+sCD14. As TRAM phosphorylation was virtually absent in unstimulated cells, this pathway may need to be activated by exogenous or endogenous stimuli.

### Kinase phosphorylation downstream of ceramide/TLR4

**Figure 3:**
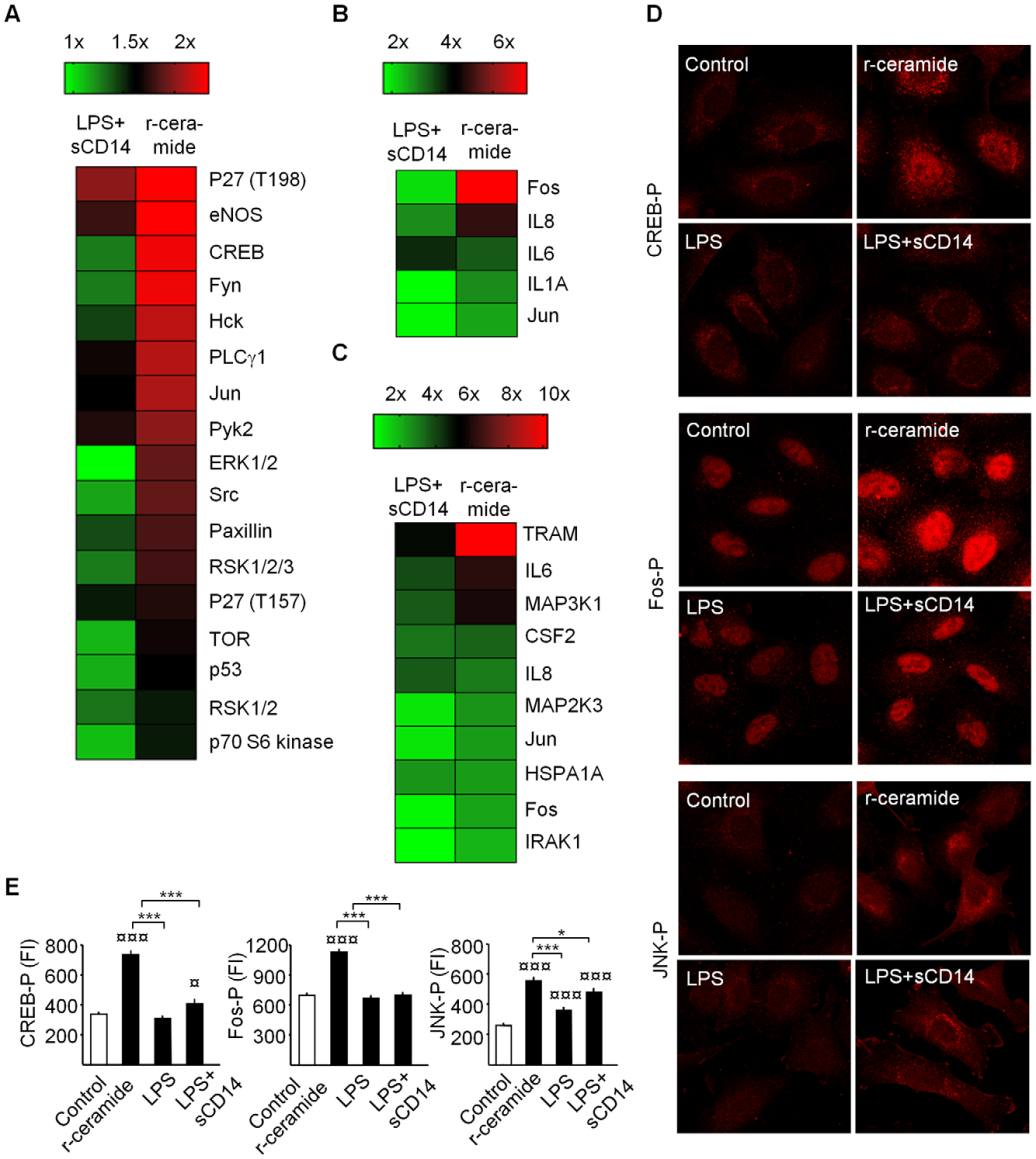
Protein phosphorylation and transcriptomic response to r-ceramide or LPS+sCD14. Panel A shows a phosphoproteomic heat map of A549 cells stimulated for 60 min with r-ceramide (SMase, 1 U/ml) or LPS+sCD14 (10+1 µg/ml) compared to untreated cells. Fold changes in protein phosphorylation levels were determined, using the Human Phospho-Kinase Array Kit. Panels B and C show gene expression heat maps of human epithelial cell RNAs. A549 or A498 cells were stimulated for 1 h or 3 h with r-ceramide or LPS+sCD14. Panels D and E show CREB phosphorylation (CREB-P) activated by r-ceramide but not by LPS+sCD14 (rabbit anti-human CREB-P primary antibodies). Fos-P activated by r-ceramide but not by LPS+sCD14 (rabbit anti-human Fos-P primary antibodies). JNK phosphorylation (JNK-P) by r-ceramide and LPS+sCD14 (rabbit anti-human JNK-P primary antibodies). Fluorescence intensities were quantified with LSM 510 software. Mean ± SEM, n = 60 cells/treatment, * p<0.05, *** p<0.001, compared to LPS+sCD14 or ¤ p<0.05, ¤¤¤ p<0.001 compared to unstimulated cells.

To define signaling downstream of ceramide/TLR4 and TRAM, we examined kinase phosphorylation, using phosphoarrays specific for 46 protein kinases and substrates (Figure 3A). Ceramide release stimulated the phosphorylation of twelve protein kinases: p27^T198^, eNOS, CREB, Fyn (all 2.3-fold), Hck, PLCγ1, Jun (all 2.1-fold), Pyk2 (2-fold), ERK1/2 and Src (1.9-fold), RSK1/2/3 (1.8-fold), p27^T157^ (1.7-fold), and p53 (1.6-fold). Antibacterial effectors included eNOS, which regulates nitric oxide and related antibacterial effector functions [34] and Hck, a Src-family tyrosine kinase associated with secretory lysosomes in neutrophils and phagosome-lysosome fusion [35]. A number of the significantly phosphorylated proteins activate IRF3- and AP1-dependent transcription. PLCγ1 catalyzes the formation of inositol 1,4,5-trisphosphate and diacylglycerol from phosphatidylinositol 4,5-bisphosphate, leading to PKC activation and CREB (cAMP response element binding) phosphorylation [36], [37]. CREB is then phosphorylated and binds to CBP (CREB-binding protein), which preferentially associates with phosphorylated IRF3 [38], [39], leading to IRF3. Fyn is a Src family tyrosine kinase implicated in the activation of PKA, a protein kinase involved in CREB phosphorylation [40]. Jun in combination with Fos bind to and are a part of the AP-1 transcription factor complex [41], which induces the transcription of proinflammatory cytokines. Pyk2 activation is highly correlated with the stimulation of c-Jun N-terminal kinase (JNK). Identified phosphorylation targets also included ERKs (ERK1/2, extracellular signal-regulated kinases) which activate downstream protein kinases and transcription factors, including IRF3 and AP-1 [42].

CREB phosphorylation in r-ceramide-activated cells was confirmed by confocal microscopy (Figure 3D, E, p<0.001 compared to control), but was not detected in LPS-stimulated cells. We obtained similar results using antibodies specific for phosphorylated Fos (Figure 3D, E). JNK phosphorylation, in contrast, was similar after r-ceramide and LPS+sCD14 stimulation (Figure 3D, E), suggesting that JNK signaling was not ceramide-specific (p<0.001 compared to the control). The results suggest that ceramide-induced TLR4 signaling causes rapid phosphorylation/transcription of proteins involved in IRF3 and AP-1 transcription, including CREB, Fyn, PLCγ, MAP kinases, ERK1/2 and Fos/Jun (Figure S4). LPS+sCD14, in contrast, caused a weaker phosphorylation response, comprising p27^T198^ (2-fold), eNOS (1.8-fold), PLCγ1 (1.7-fold), Pyk2 (1.7-fold), and Jun (1.6-fold), but not the remaining targets that were phosphorylated in response to r-ceramide (Figure 3A).

### Transcriptional activation in response to ceramide

Innate immune activation in response to ceramide was further examined by TLR SuperArrays and compared to LPS+sCD14 (Figure 3B, Figure S5). After one hour, five genes in A549 cells had responded to r-ceramide: Fos (6.5-fold) and Jun (2.1-fold), IL-8 (4.4-fold), IL-6 (2.9-fold) and IL-1α (2-fold). The response showed a similar, restricted repertoire in A498 carcinoma cells after three hours (Figure 3C). r-Ceramide upregulated TRAM, Fos and Jun transcription (10.2-, 2.2- and 2.4-fold, respectively). Ceramide activated IL-6 transcription (6.2-fold), MAP3K1 and MAP2K3 (5.9- and 2.5-fold), as well as IL-8 and CSF2 transcription levels (about 3-fold). In contrast, LPS+sCD14 did not significantly stimulate Fos (1.5-fold), Jun (1.2-fold) or IL-1α after one hour. After three hours, only IL-8 transcription was higher in response to LPS+sCD14 than to r-ceramide. The transcriptional profile confirmed the difference between ceramide and LPS+sCD14 activated cells, consistent with a different transcription factor usage.

### IRF3 translocation to the nucleus in response to ceramide/TLR4

**Figure 4:**
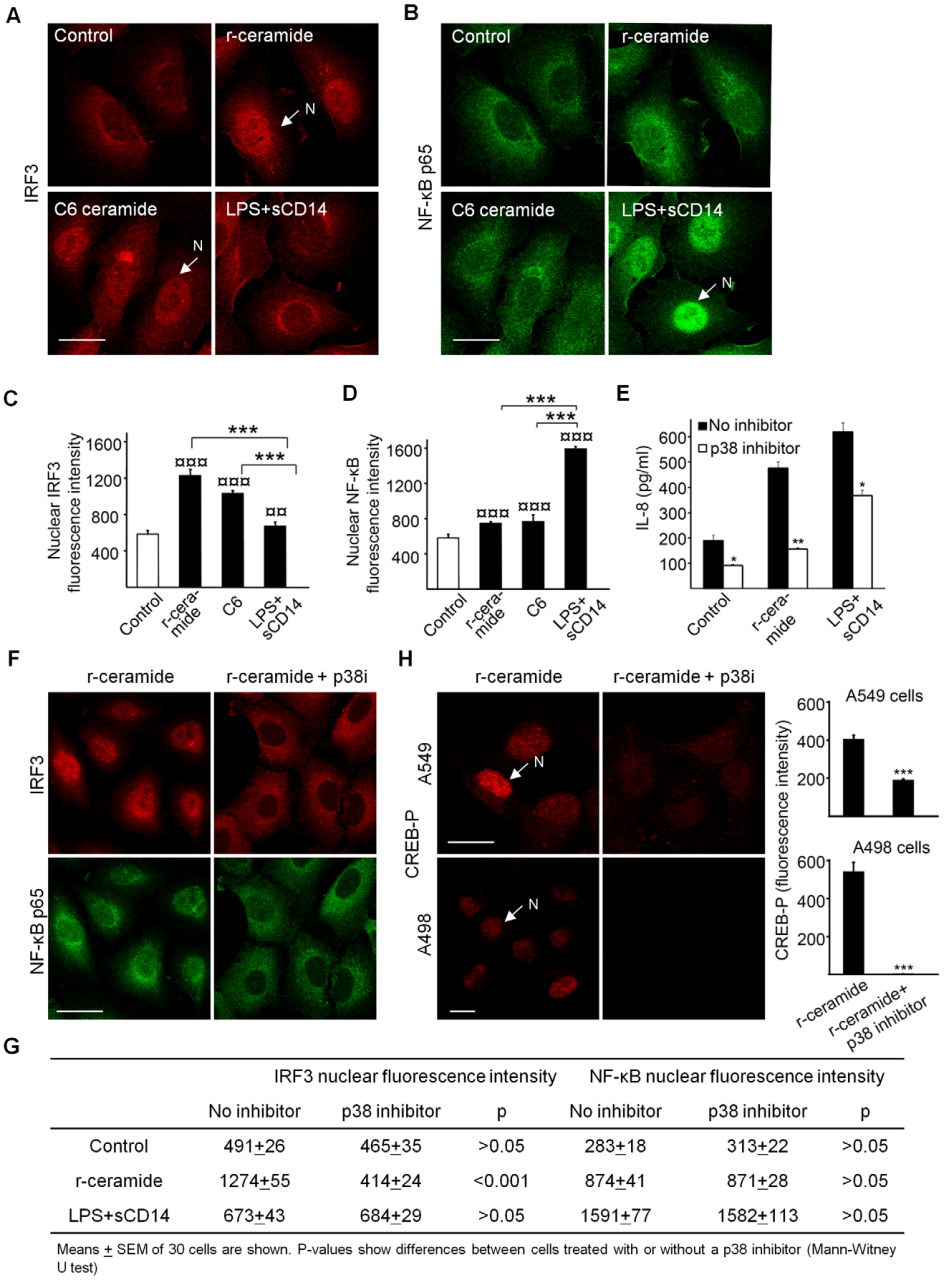
Nuclear IRF3 translocation in response to ceramide/TLR4. Panels A and C show IRF3 and panels B and D show NF-κB p65 translocation in 70% confluent A549 cells exposed to r-ceramide (SMase (1U/ml), C6 ceramide (30 µg/ml) or LPS+sCD14 (10+1 µg/ml) for 90 min (mean ± SEM of 50 cells/sample, *** p<0.001 between different stimulations, ¤¤ p<0.01, ¤¤¤ p<0.001 compared to control). Panel E shows that p38-MAPK inhibition reduces the IL-8 response to r-ceramide and panels F and G show that p38-MAPK inhibition reduces the nuclear translocation of IRF3 but not NF-κB in A549 cells. Panel H shows that CREB phosphorylation is reduced by p38 inhibition in A549 and A498 cells pre-treated with SB202190 (30 min, 20 µM) and stimulated with r-ceramide (SMase (1 U/ml).

IRF3 is an interferon regulatory transcription factor and following TLR4 activation, phosphorylated IRF3 homodimers translocate from the cytosol to the nucleus [43], [44], [45], [46]. By confocal microscopy (Figure 4A, C) we observed that r-ceramide triggered IRF3 translocation to the nucleus (p<0.001 compared to unstimulated cells, 90 min). We confirmed the results in a human bladder epithelial cell line (J82, Figure S6A) in which ceramide release caused rapid IRF3 translocation. In cells exposed to LPS+sCD14, the nuclear IRF3 translocation was weak (p>0.05 compared to control) and fewer dimers were formed after exposure to LPS+sCD14. In the bladder epithelial cells, the IRF3 response to LPS+sCD14 or LPS alone was low. LPS+sCD14-induced NF-κB p65 translocation, but the NF-κB response to r-ceramide or C6 ceramide was weak (Figure 4 B, D). For a broader field of view see Figure S6B.

Signaling through p38 MAPK has previously been shown to stimulate proinflammatory responses, including IL-8, IL-6 and TNF [47], [48], [49]. The activation of MAP3K1 and MAP2K3 by r-ceramide exposure of A549 cells suggested that this pathway might be involved upstream of IRF3. Pretreatment of the cells with a p38 inhibitor (SB202190) reduced the IL-8 response to r-ceramide (Figure 4E) and prevented nuclear translocation of IRF3 (Figure 4F, G). NF-κB p65 translocation was not affected by the p38 inhibition (Figure 4F, G). The results suggest that ceramide/TLR4 activates IRF3- rather than NF-κB-dependent transcription, and that the IRF3 response involves p38 MAPK-dependent mechanisms. CREB-phosphorylation was also markedly reduced after p38 inhibition, as shown by confocal microscopy (>99% in A498 cells, >50% in A549 cells, Figure 4H) and Western blots (Figure S7).

Previous work has suggested that the phosphorylation of TRAM is mediated by PKC-epsilon, which is activated downstream of TLR4 [50]. Given that PKC-epsilon is also essential for IRF3 activation, this pathway was examined, using the pan-PKC inhibitor Bisindolylmaleimide II. The inhibitor reduced the response to PMA, which was used as a PKC dependent, positive control. In contrast, the response to ceramide was not impaired (Figure S8).

To further examine the relationship of the ceramide/TLR4 pathway to the classical IRF3 activation pathway, cells were transfected with TBK1 siRNA and responses were compared to irrelevant siRNA transfected cells. In parallel, the cells were transfected with TLR4 and TRAM siRNAs (Knock down efficiency for TLR4 and TRAM was >90% and 64% for TBK1, Figure S9). IRF3-P responses to r-ceramide were reduced by the TLR4 and TRAM siRNAs but were less affected by suppression of TBK1 expression. The response to LPS+sCD14 showed a similar pattern (Figure S9).

These results suggest that the pathway of IRF3 activation identified here has several new features, including p38 dependence and PKC independence. The involvement of TBK1 needs further study.

### Bacterial fimbriae and IRF3 translocation in infected human renal tubular epithelial cells

**Figure 5:**
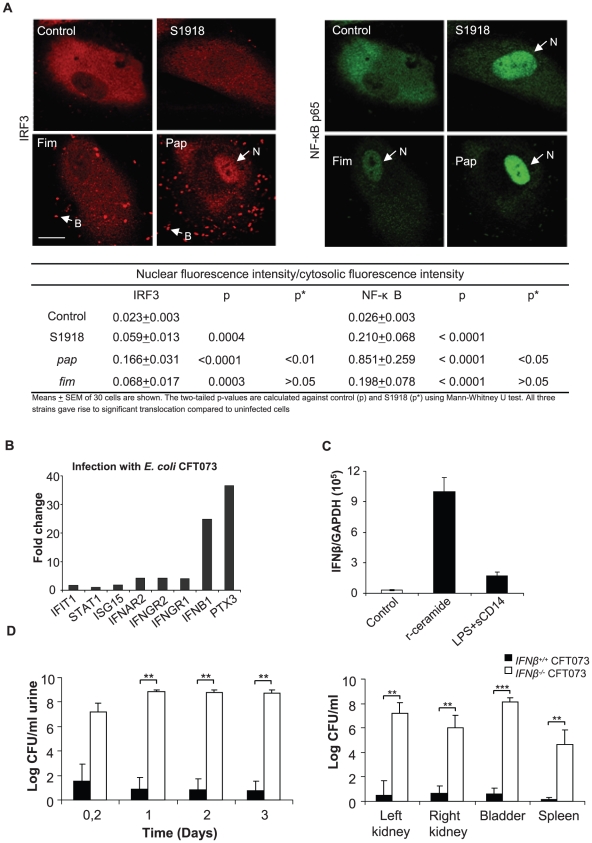
Nuclear IRF3 translocation after stimulation with P-fimbriated *E. coli* and the importance of IFN signaling for host resistance against UTI. Panel A shows nuclear translocation of IRF3 and NF-κB in human kidney cells infected with P-fimbriated *E. coli* (10^9^ CFU/ml, for 90 min). *E. coli* S1918 was transformed with recombinant plasmids pPIL 110-75 encoding P-fimbriae, (S1918*pap)* or PKL-4 encoding type 1 fimbriae (S1918*fim*). Infection increased nuclear staining above background for all three strains. *E. coli* S1918*pap* triggered stronger IRF3 and NF-κB responses than *E. coli* S1918 or *E. coli* S1918*fim*. Quantifications of nuclear fluorescence intensity are given in the table, means ± SEMs of 30 cells/sample; for a broader view see Figure S11. Panel B shows IRF3 target gene expression after stimulation of A498 kidney epithelial cells with *E. coli* CFT073 (Illumina whole genome microarrays, figure shows fold change above uninfected control cells, ≥log 2 cut off). Infection stimulated genes in the IFN pathway and increased the IFNβ response. Panel C shows TLR-specific superarray data. Panel D shows the increased susceptibility of *Ifnβ^−/−^* mice to infection with CFT073, compared to wt mice. Bacterial numbers are compared in urine and tissues (Kidneys, bladders and spleens).

To examine if the IRF3 response is triggered in a pathogen-specific manner involving P fimbriae, we stimulated primary cultures of human CD14+ renal tubular epithelial cells (HRTEC) with isogenic P-fimbriated (*E. coli* S1918*pap*) or type 1-fimbriated (*E. coli* S1918*fim*) *E. coli* strains and examined IRF3 by confocal microscopy. Non-fimbriated *E. coli* S1918 was used as a control. *E. coli* S1918*pap* induced higher nuclear IRF3 translocation and IRF3 phosphorylation than *E. coli* S1918, consistent with results in ceramide-stimulated cells (Figure 5A and Figure S10, p<0.01 for a broader view). There was less IRF3 translocation in response to *E. coli* S1918*fim* or to the non-fimbriated control *E. coli* S1918. All three strains stimulated an NF-κB response, but NF-κB translocation was higher in cells infected with P-fimbriated *E. coli* compared to type 1-fimbriated *E. coli* (Figure 5A, p<0.05). Uninfected cells showed no evidence of nuclear IRF3- or NF-κB translocation. The same phenomenon was observed in A498 kidney epithelial cells (Figure S11). In addition, preliminary Western blot analysis of IRF3P in infected cells suggested that S1918pap and S1918*fim* stimulated a higher response than S1918 (Figure S11).

### Increased susceptibility to UTI in *Ifnβ^−/−^* mice

IRF3 target gene expression was examined by microarray analysis. We infected A498 kidney epithelial cells *in vitro* with virulent CFT073 or non-pathogenic *E. coli* (4 hours, 10^8^ CFU/ml) and complementary RNA was hybridized to Illumina whole genome microarrays. There was a dramatic IFNβresponse to infection (24-fold above uninfected control cells, ≥log 2 cut off) (Figure 5B). By Ingenuity Pathway analysis, we detected significant activation of several members of the interferon-signaling pathway such as *IFIT1*, *STAT1 ISG15*, *IP-10* and *IFNAR2*. A weaker ISG15 response was observed (1.8-fold above background). By RT-PCR, a strong IFNβ response to r-ceramide was confirmed in human kidney cells (A498, Figure 5 B and C).

To examine if the effects of IRF3 on host susceptibility are IFNβ-dependent, we infected *Ifnβ^−/−^* mice with *E. coli* CFT073 and examined parameters of disease and bacterial persistence (Figure 5E). Bacterial clearance was drastically impaired in *Ifnβ^−/−^*mice compared to wt controls (Figure 5E, p<0.001 between *Ifnβ^−/−^*and wt mice in urine, kidneys and bladders). The *Ifnβ^−/−^* mice also developed abscesses. Positive spleen cultures confirmed systemic spread of infection in these mice, which also developed symptomatic disease and were sacrificed on day three (Figure 5E). The results suggest that IFNβ is activated by infection and that IFNβ might be an essential effector molecule in IRF3-dependent bacterial clearance.

### IRF3 promoter polymorphisms in patients with urinary tract infection

To examine if UTI susceptibility is associated with differences in *IRF3* promoter efficiency, *IRF3* promoter sequence variation was studied in two highly UTI-prone patient populations. Sample 1 comprised children in southern Sweden, with a consistent UTI pattern over several years: either severe recurrent kidney infections (APN; n = 21) or asymptomatic carriage of *E. coli* with no prior symptomatic infection (primary asymptomatic bacteriuria, ABU, n = 16). These children were identified after prospective, long-term follow-up of a larger patient group. Sample 2 comprised adults in western Sweden, with a history of childhood UTI (n = 82). They were enrolled in a prospective study of febrile UTI (APN) in the 1970s and were recently re-evaluated, after about 30 years, to investigate UTI morbidity and long-term effects on health and kidney function. Both samples included additional patients who developed ABU secondary to an APN episode (secondary ABU, n = 16 in sample 1 and n = 61 in sample 2). Controls were children without UTI or related morbidity (n = 27) and adult blood donors (n = 62) from the same areas.

**Figure 6:**
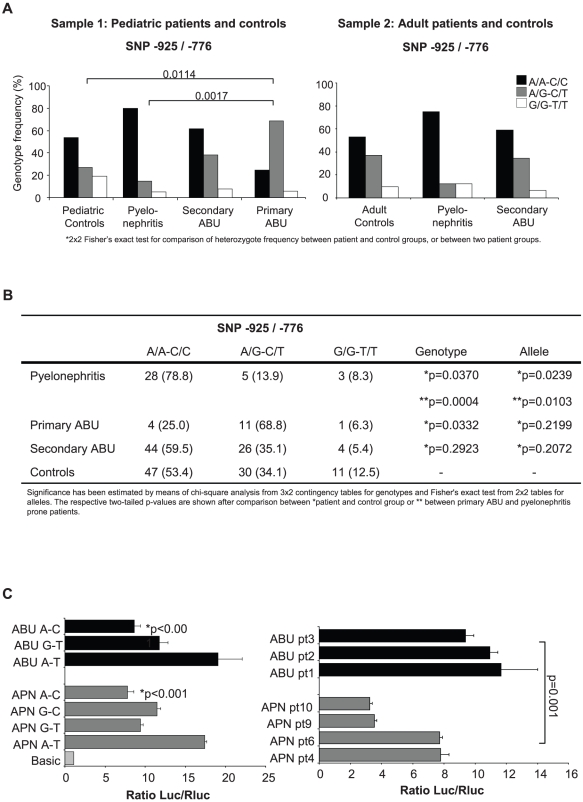
Human *IRF3* promoter sequence variation and activity in UTI-prone patients. Panel A shows the *IRF3* promoter polymorphisms (−925 and −776) in highly selected, UTI-prone pediatric and adult patients compared to healthy controls. The homozygous, A/A, C/C genotype at positions −925 and −776 dominated in pyelonephritis-prone patients, while the G/A, C/T heterozygous genotype was more common in the asymptomatic carriers. Panel B presents genotype and allele counts for markers −925 and −776 with samples 1 and 2 combined. Panel C shows the *IRF3* promoter activity in transfected human kidney epithelial cells. *IRF3* promoter SNPs representing the ABU and APN genotypes were amplified from patient DNA altered by site-directed mutagenesis and cloned into a luciferase reporter plasmid. The predominating APN genotype (A-C) lowered the luciferase expression levels compared to the ABU genotype (A-T). Graphs show means of one representative experiment out of three, each done in triplicate.

DNA sequencing of *IRF3* promoters from UTI patients revealed variation at the −925 and −776 positions. SNPs −925 and −776 were linked in the study population (r^2^ = 1.0) but the *IRF3* genotype varied with UTI severity (Figure 6A–B). Genotype counts for −925 and −776 were in Hardy Weinberg Equilibrium across both case and control samples apart from the APN group (χ^2^ = 47, p<0.001), indicating effects of genetic drift in the APN group. We observed significant differences for the two studied markers between cases and controls in allelic or genotypic models. In sample 1, most of the APN patients were homozygous for the two positions (A/A–C/C, 79% vs. 25% in primary ABU, Figure 6A p = 0.0017). The results in APN patients were confirmed in sample 2, with 75% homozygous and 13% heterozygous SNPs compared to 53% and 37% in adult controls. The differences were confirmed when the two samples were combined, as shown in Figure 6B. Furthermore, the minor allele frequency was decreased in APN compared to primary ABU (p = 0.0103) and controls (p = 0.0239) (Figure 6B). The minor allele frequency for paediatric UTI patients, adult UTI patients and the relevant controls are demonstrated in Supplemental Table S4 in Supporting Information S1.

The *IRF3* genotype of the secondary ABU patients resembled the APN groups in both samples, consistent with their prior APN episodes.

### The acute pyelonephritis-associated promoter SNPs reduced transcription efficiency

To examine if the *IRF3* promoter variation influences transcription efficiency, we cloned promoters from one patient with APN and one with ABU into a luciferase reporter vector. We then changed the APN haplotype at positions −925, −776 to the predominating ABU haplotype (A-C to G-T), or the ABU haplotype to the APN haplotype (G-T to A-C) by site-directed mutagenesis. We then transfected A498 human kidney epithelial cells with the different promoter constructs and determined luciferase activity (Figure 6C). The promoter was functional in these cells, resulting in luciferase activity above the vector control.

Transcriptional activity from the APN promoter (A-C) was about 50% lower compared to the ABU (A-T) promoter (Figure 6C). This difference could be attributed to the polymorphic sites, as the promoter activity increased when the APN A-C haplotype was mutated to G-T and decreased when the ABU G-T haplotype was mutated to A-C (p<0.001). This difference was confirmed by cloning the *IRF3* promoters from three additional APN (A-C) and three ABU (G-T) patients (p = 0.001). The results show that the *IRF3* promoter efficiency is reduced by the SNPs occurring in about 80% of APN patients, consistent with the human SNPs reducing IRF3 expression and increasing the risk for APN.

## Discussion

IRF3 was originally described as a transcription factor controlling interferon responses to viral infection [51]. More recently, the involvement of IRFs in antibacterial defense and immunoregulation by TLRs has received more attention, since NF-κB, IRF3 and AP-1 form transcriptional complexes that regulate innate immune responses in monocytes [52]. The relevance of IRF3 to human pathology has not been investigated, however. We show that IRF3 is activated in a pathogen-specific manner by P-fimbriated, uropathogenic *E. coli*, through a new signaling pathway involving TLR4, TRAM, CREB and p38. In the absence of IRF3, acute morbidity and extensive tissue damage are dramatically augmented, consistent with the need for this pathway to maintain a functional antimicrobial defense. Host susceptibility to common infections like UTI may thus be strongly influenced by single gene modifications affecting the innate immune response.

Mucosal pathogens exploit the extracellular domains of sphingolipids as receptors for AB toxins such as Shiga and cholera toxin, as well as attachment ligands for *Pseudomonas aeruginosa*, HIV gp120 and uropathogenic *E. coli* [53], [54], [55]. This study provides evidence that P-fimbriated *E. coli*, SMase and exogenous, free ceramide all activate the IRF3-dependent innate immune response. Soluble, exogenous ceramide and SMase were used in parallel in these experiments to ensure that the synthetic, short-chained form of ceramide and the endogenous, long-chained form adequately represented the membrane-anchored species in intact cells. SMase is contaminated by low amounts of LPS, but these trace amounts were insufficient to activate an innate immune response in the CD14-negative mucosal cells used in this study [9], [14]. FRET analysis showed that ceramide is approximated to TLR4 in the cell membrane, suggesting that a direct interaction with TLR4 and/or the early adaptors trigger this pathway. Such ceramide-induced TLR4/IRF3 signaling might offer a general mechanism for host sensing of pathogens that perturb membrane sphingolipids in mucosal cells.

To provide further evidence that ceramide signaling via TLR4 to IRF3 discriminates virulent from commensal bacteria, we infected *Irf3^−/−^* mice with a commensal-like *E. coli* strain from a patient with asymptomatic bacteriuria. This strain did not trigger a significant response and was cleared efficiently, suggesting that the IRF3 pathway was not alerted. In constrast, a P-fimbriated transformant triggered rapid, septic infection in the *Irf3^−/−^* but not in wt mice linking this virulence factor that recognizes glycosphingolipid surface receptors, to the IRF3-dependent host response. In contrast, IRF3 was not activated by a type 1 fimbriated isogenic strain, suggesting a preference for glycosphingolipid rather than glycoprotein receptors. This does not negate the previous finding that FimH acts as an immune inducer, protecting against viral infection associated with TLR4 and type 1 interferon signaling, but suggests that the mechanisms differ [56]. The results confirm the pathogen specificity of the IRF3 response and the role of P fimbriae as a virulence ligand triggering this response. As a consequence of this selective IRF3 activation, the uropathogenic or P-fimbriated, commensal *E. coli* strains influenced epithelial gene transcription in a pathogen-specific manner.

IRF3 phosphorylation in response to ceramide was controlled by TLR4 and TRAM, as shown using specific siRNA knock down. Activation was not TBK1 or PKC dependent, however, suggesting alternative activation compared to previously described mechanisms of IRF3 activation [50]. A schematic of the identified kinases and targets is given in supplemental Figure S4. Although this signaling pathway has not been entirely deciphered, a strong involvement of TRAM and CREB was detected as well as involvement of p38 MAPK-dependent events. In this model, IRF3 activation was not controlled by PKC dependent mechanisms, however. The involvement of TBK1 is not clear, but preliminary experiments did not provide evidence that TBK1 controlled IRF3 phosphorylation in this pathway. In addition, P-fimbriated *E. coli* strains and ceramide significantly activated NF-κB, thus providing a broad basis for the innate immune response to the intact, complex pathogen. Importantly, the IRF3 response differed after LPS+sCD14 stimulation, further suggesting that pathogen recognition and pattern recognition agonists trigger partially different signaling pathways.

The phenotype of *Irf3^−/−^* mice predicted that reduced IRF3 expression could also increase human susceptibility to severe kidney infection. In support of this hypothesis, there were marked promoter sequence differences between children with ABU or APN in a long-term prospective study and we confirmed an association of polymorphisms to disease severity in adult patients who were followed for about 30 years after their first febrile UTI episode. In the past, we have shown that genetic variation affecting innate immunity modifies human UTI susceptibility [2]. Chemokine receptor expression and neutrophil function are modified by CXCR1 expression, and promoter variants reducing TLR4 expression are coupled to asymptomatic bacteriuria [22], [57], [58], [59], [60]. The present study adds IRF3 to this short list of polymorphic innate immune response genes that distinguish asymptomatic carriers from APN-prone patients.

The human *IRF3* promoter has a number of transcription factor binding sites, including a HOX box, three SP1 sites, NF1, USF, SRF and IRF1-like site and functional elements are within a 113-nucletide long fragment, containing one Sp-1 site, the IRF1-like site, NF1 and HOX box. SNP −925 is located within this region, indicating a possible role in promoter efficiency. *IRF3* promoter SNPs were first described in patients with systemic lupus erythematosus (SLE) [61]. It was speculated that the A-C haplotype increased *IRF3* transcription and that the G-T haplotype might protect against SLE by reducing type I IFN production. The effect of the *Irf3* deletion on disease susceptibility in mice suggested, however, that risk might be associated with reduced, rather than increased, IRF3 function. This idea was supported by luciferase reporter assays designed to test the *IRF3* promoter sequences typical of APN- or ABU-prone individuals.

UTIs are among the most common bacterial infections in man, and remain a major cause of morbidity and mortality [62], [63]. A subset of disease-prone individuals is at risk for recurrent severe pyelonephritis and renal dysfunction. Therefore, there is a need to identify and treat these patients, preferably in infancy, when many of them experience their first febrile UTI episode. Although predictive diagnostic tools have been suggested [58], [59], [60], the present study identifies *IRF3* for the first time as an innate immune response gene involved in UTI. Thus, *IRF3* may be a new molecular target in the diagnosis of UTI susceptibility, potentially creating more precise approaches for detection and prevention of severe, recurrent kidney infection and associated debilitating morbidity.

## Methods

### Ethics statement

For research involving humans, informed written consent was obtained from all participants or their parents/guardians. The study was approved by the Ethics Committee of the medical faculty, Lund University, Sweden (LU106-02, LU236-99).

All the animal experiments were performed with the permission of the Animal Experimental Ethics Committee at the Lund District Court, Sweden (numbers M166-04 and M87-07). Experimental UTI was performed in a level P2 biohazard laboratory within the MIG animal facility and was governed by the following directive, law, ordinance and provisions: Council Directive EG 86/609/EEC, the Swedish Animal Welfare Act (Djurskyddslag: 1988:534) and the Swedish Animal Welfare Ordinance (Djurskyddsförordning: 1988 :539). Provisions regarding the use of animals for scientific purposes: DFS 2004:15, DFS 2005::4, SJVFS 2001:91, SJVFS 1991:11.

### Reagents

SMase (*Staphylococcus aureus*), bovine serum albumin, SDS, LPS (*Salmonella typhimurium*), C6 ceramide, SB202190 and Bisindolylmaleimide II were from Sigma Aldrich, St Louis, MO, USA. Soluble CD14 (sCD14) was from Biometec, Greifswald and IL-8 was quantified by Immulite 100, Siemens, Germany. Lipofectamine 2000 transfection reagent was from Invitrogen. siRNA downregulation (Supplemental Table S1 in Supporting Information S1) was validated by qRT-PCR, using primers: TLR4 (Hs00152939, Applied Biosystems), MyD88 (QT00203490, Qiagen), TRAM (QT00033341, Qiagen), TBK1 (QT00078393). TBK1 siRNA (sc-39058), a pool of 3 target-specific 19–25 nt siRNAs was from Santa Cruz Biotechnology (USA). Human Phospho-Kinase Array Kit ARY003 was from R&D Systems, Abingdon, Oxford, UK. Transcriptome analysis of r-ceramide or LPS+sCD14 activated cells (1 or 3 hours) was by Superarray (PAHS018, SaBioscience) for 84 TLR signaling pathway genes. *IRF3* promoter SNPs were identified by Pyrosequencing using the PSQ 96 SNP Reagent Kit (Biotage, Uppsala, Sweden, Supplemental Table S1 in Supporting Information S1). Rabbit anti-human TLR4 primary antibodies were from eBioscience, CA, USA, mouse anti-ceramide primary antibodies, clone MID 15B4 from ALEXIS Corporation, Lausen, Switzerland. Rabbit anti-human primary antibodies against CREB-P (Ser 133), Fos-P (Thr 232), JNK-P (Thr 183/Tyr 185) and IRF3 and mouse anti-human-NF-κB p65 antibodies from Santa Cruz Biotechnology (USA), rabbit anti-IRF3-P (Ser 396) antibodies were from Cell Signaling Technology. Rabbit anti-human-TRAM-P (raised against the N-terminal end of the protein, aa 7–21 containing phosphoSer at aa 16) and TRAM antibodies were from FabGennix Inc., Frisco, TX, USA, NIMP-R14 rat anti-mouse neutrophil specific antibodies from Abcam, Cambridge, USA, polyclonal rabbit antiserum raised against a peptide within the PapG adhesin (CRPSAQSLEIKHGDL) was used to detect P-fimbriated *E. coli*. Alexa 488 anti-rat IgG, Alexa 568 anti-rabbit IgG, Alexa 568 anti-mouse IgM and Alexa 488 anti-mouse-IgG secondary antibodies were from Invitrogen, Eugene, Oregon, USA. Swine anti-rabbit immunoglobulins-HRP secondary antibodies were from DAKO A/S, Glostrup, Denmark and Santa Cruz Biotechnology (USA). FRET and fluorescence microscopy was by LSM510 META confocal microscope (Carl Zeiss, Oberkochen, Germany).

### Cell cultures

The human lung adenocarcinoma A549 (ATCC CCL-185) and kidney carcinoma A498 (ATCC HTB-44) epithelial cell lines were grown in RPMI 1640 supplemented with 1 mM sodium pyruvate, 1 mM non-essential amino acids, 50 µM/ml gentamicin, and 5% FBS. Human renal tubular epithelial cells (HRTEC) were isolated as described [64]. Cells were maintained at 37°C+5% CO_2_ in a humidified atmosphere, split weekly and exposed to P-fimbriated, type I fimbriated or non-fimbriated *E. coli*, 0.1–1 U/ml of SMase, freshly prepared C6 ceramide or LPS+sCD14. IL-8 secretion was quantified by Immulite 100 (Siemens, Bad Nauheim, Germany).

### FRET

LSM 510 Meta confocal laser-scanning microscopy was used for FRET acceptor photobleaching and imaging of epithelial cells. Cell stimulation/infection was in 8-well chamber slides (LabTek, Nunc, RPMI+5% foetal calf serum). The cells were first stimulated with r-ceramide (1U of SMase/ml) or LPS+sCD14 (10+1µg/ml), fixed with 3.7% formaldehyde and stained with a mouse antibody specific for native ceramide (sphingosine-trans-D-erythro-2-amino-4-octadecene-1.3-diol) and with a rabbit polyclonal antibody, specific for the extracellular domain of TLR4 (amino acids 6–169). Secondary antibodies to TLR4 and free ceramide were conjugated with Alexa-488 (donor) and Alexa-568 (acceptor), respectively. FRET efficiency was estimated in percent of fluorescence increase calculated by: FRET efficiency = ((IDA-IDB)/IDA)×100% where IDA is the donor intensity after bleaching and IDB the donor intensity before bleaching.

### siRNA transfection

A549 human epithelial cells in 24-well plates (TPP) were siRNA transfected, using Lipofectamine 2000 (Supplemental Table S1 in Supporting Information S1). Knockdown efficiency was validated by qRT-PCR. After a 72 h incubation, transfected cells were stimulated with SMase (1 U/well) or LPS+sCD14 (10+1µg/ml). Supernatants were collected after 24 h.

### RT-PCR array

Total extracted mRNAs were converted to cDNA using RT^2^ First Strand Kit (SABioscience Corporation, Fredrick, MA, USA). The transcriptomic profile of cells exposed to r-ceramide or LPS+sCD14 was examined using a RT-PCR-based superarray, containing 84 genes involved in TLR signaling (SABiosciences, PAHS018). Gene expression levels were calculated by the ΔCt method and normalized to five housekeeping genes. RT-PCR was used to determine the efficiency of siRNA knockdown. The TaqMan system was used to quantify TLR4 and GAPDH cDNA and the QuantiTect SYBR Green systems was used to quantify other genes of interest. cDNA was quantified by RT-PCR using a Rotor gene 2000 instrument (Corbett Life Science, Sydney, Australia) and normalized against GAPDH.

### Phospho-kinase array

Protein phosphorylation was quantified using the Human Phospho-Kinase Array Kit (Proteome Prolifer Array, R&D Systems, Abingdon, Oxford, UK). Protein extracts were prepared from 100% confluent A549 cells cultured in 6-well plates and treated with 1U/well SMase or LPS+sCD14 (10+1µg/ml). Untreated cells were used as control. The signals were detected with the ECL Plus Western Blotting Detection System (GE Healthcare).

### SDS-PAGE and immunoblotting

In order to detect phosphorylated TRAM, A549 cells grown in 6-well plates were stimulated with 1U/mL SMase, 0.1 µg/mL LPS+sCD14 (10+1µg/ml), 15 µg/mL C6 (Sigma) or RPMI medium alone for 45 and 90 min. Cells were lysed in ice-cold buffer (10 mM HEPES-KOH, 5 mM EDTA, 0.5% Nonidet P-40 and 10 mM KCl, pH 7.9) containing a protease inhibitor mix (Complete, Roche Diagnostics GmbH, Mannheim, Germany) and 1mM Na_3_VO_4_. After 10 min incubation, lysates were centrifuged for 5 min at 12000 g at 4°C and protein concentrations in the collected supernatants were quantified using the DC protein assay kit (Bio-Rad, Hercules, USA). Proteins in the lysates were separated by SDS-PAGE (4–12% NuPAGE Bis-Tris gels, Invitrogen) on ice with NuPAGE MES SDS running buffer (Invitrogen). Proteins were transferred to a polyvinylidene difluoride (PVDF) membrane using NuPAGE transfer buffer (Invitrogen) and the membrane was probed with phospospecific primary antibodies followed by HRP-labeled, swine anti-rabbit IgG. Bound antibodies were visualized with the ECL Plus Western Blotting Detection System.

### Native PAGE and immunoblotting

IRF3 dimerization was detected by native PAGE and immunoblotting. A549 cells cultured in 6-well plates were stimulated with medium alone, 1 U/mL SMase and 0.1 µg/mL or LPS+sCD14 (10+1µg/ml), for 90 min. Whole cell lysates in a buffer containing 50 mM Tris HCl, pH 7.5, 400 mM NaCl, 1mM EDTA, 1% Nonidet P-40 were separated by electrophoresis on a 7.5% native Tris-glycine gel [65]. Membranes were incubated with primary antibodies against human IRF3 (Fl-425, Santa-Cruz) diluted 1∶1000 and anti-rabbit IgG-HRP (1∶1000). IRF3 monomers and dimers were detected with the ECL Plus Western Blotting Detection System.

### DNA microarray analysis

In brief, A498 cells (n = 350000) were seeded in 6-well plates and infected with CFT073 (10^8^ CFU/ml), total RNA was extracted (Trizol, Invitrogen, USA) and cleaned by a Qiagen RNeasy. RNA was reverse-transcribed to biotin-labeled cRNA using a TargetAmp Nano-g Biotin-aRNA Labeling kit (Epicentre Biotechnologies, Madison, USA). Labeled cRNAs were hybridized onto an Illumina HumanHT-12 Expression Beadchip for 16 hours at 58°C. The arrays were then washed and stained (Illumina Wash Protocol) and scanned using a BeadArray Scanner 500GX. The background-subtracted data were pre-processed to correct negative and non-significant intensities. Pre-processed data was normalized using the cross-correlation [66] and genes with a log fold change of 2 were identified as differentially expressed. Data was preprocessed using RMA implemented in the free software packages R and Bioconductor (http://www.r-project.org). For more details, see Yadav et al.

Differentially expressed genes were categorized using the Functional Annotation Clustering Tool in the Database for Annotation, Visualization and Integrated Discovery (DAVID) [67] and the EASE score (a modified enrichment score derived from Fisher exact P-value) was used to judge the enrichment. To further study signaling pathways altered by CFT073, the differentially expressed genes were submitted for Ingenuity Pathway Analysis (Ingenuity Systems, Redwood City, CA).

### Bacteria and growth conditions

*Escherichia coli* S1918 [68] (KanR) lacks genes encoding known adhesins and was used as a recipient strain for recombinant plasmid pPIL 110-75 (AmpR) carrying the papAD1100 gene cluster (pap+) [69] or PKL-4 carrying the entire fim gene cluster from *E. coli* PC31 (type I+) [68]. The *papGX* deletion mutant of *E. coli* strain 83972 (ABU 83972Δ*papGX*) was generated using the lambda red homologous recombination technique [70]. Clones with the reconstituted *pap* determinant were screened using PCR and verified by DNA sequencing. Primers used for reconstitution of a functional *pap* gene cluster in *E. coli* 83972 are shown in Supplemental Table S5 in Supporting Information S1. The growth rates of the reconstituted mutant strain and the 83972 wild type strain were shown to be identical. In addition, the ability of the reconstituted mutant to agglutinate sheep blood erythrocytes was also confirmed by agglutination assays.

### Experimental urinary tract infection (UTI)

Mice were bred at the MIG animal facilities, Lund, Sweden. Female C57BL/6 wild type or *Irf3^−/−^* (from T. Taniguichi) and *Ifnβ^−/−^* (from F. Ivars, Lund University) mice were used at 9–15 weeks. After anesthesia (Isofluorane), mice were infected by intravesical inoculation with *E. coli* CFT073 (10^9^ CFU in 0.1 mL) through a soft polyethylene catheter (outer diameter 0.61 mm; Clay Adams, Parsippany, NJ, USA). Animals were sacrificed while under anesthesia, kidneys and bladders were removed and prepared for hematoxylin-eosin staining or immunohistochemistry. Viable counts in homogenized tissues (Stomacher 80, Seward Medical, UAC House, London, UK) were determined after overnight growth on tryptic soy agar plates at 37°C. Urine samples collected prior to and daily after infection were cultured and recruited neutrophils were quantified in uncentrifuged urine by use of a hemocytometer.

### Histology and immunostaining

Kidney sections were examined by immunohistochemistry [71]. Tissue sections were dried and permeabilized in 0.2% Triton X-100, 5% goat normal serum (DAKO) in PBS, incubated with NIMP-R14 rat anti-mouse neutrophil specific antibodies (1∶200) and a polyclonal rabbit antiserum to the Pap G adhesin (1∶200) to detect P-fimbriated *E. coli* and to Alexa 488 anti-rat IgG and Alexa 568 anti-rabbit IgG secondary antibodies and nuclei were counterstained with DAPI (0.05 µM). After mounting, coverslipped slides were examined by fluorescence microscopy (AX60, Olympus Optical, Hamburg, Germany) at the Department of Pathology, Lund University, Sweden.

Confocal fluorescence immunocytochemistry was performed on cells grown to 70–80% confluence on 8-well chamber slides. After stimulation, cells were fixed and permeabilized with 0.25% Triton X-100, 5% FBS in PBS and incubated with primary antibodies diluted 1∶50 in 5% FBS in PBS overnight at 4°C. Alexa fluor-labeled secondary antibodies were applied for 1 hour at RT in the dark. In order to control specific staining of neutrophils and bacteria, slides were stained with only secondary antibodies (Figure S1). Slides were covered with mounting medium (M1289, Sigma) and cover glasses and the cells were examined with a LSM510 META confocal microscope.

### UTI-prone patients and pyrosequencing

The *IRF3* promoter from patients with UTI or healthy controls was sequenced (PSQ96, Biotage, Uppsala, Sweden) and examined for −925 and −776 polymorphisms [61]. Genomic DNA was extracted from heparinized peripheral blood using the QIAamp DNA Blood midi kit. More detailed descriptions of inclusion critera and diagnosis are provided in [58], [59], [72]. The *IRF3* promoter SNPs (−925; −776) were genotyped using Pyrosequencer PSQ 96 after PCR amplification of chromosomal DNA and a second biotinylated PCR for each SNP (for primers see Supplemental Table S2 in Supporting Information S1).

### Transient transfection and dual luciferase reporter assay

The promoter sequences from extracted chromosomal DNA derived from APN and ABU patients were PCR-amplified using the Infusion primers 5′ IRF3 NheI and 3′IRF3 NcoI (Supplemental Table S3 in Supporting Information S1) and Phusion hot start polymerase according to the manufacturer (Finnzymes Oy, Finland). Amplicons were introduced by recombination, using the Infusion cloning technique (Clontech), into a NheI- and NcoI-cleaved and gel-purified luciferase reporter vector, pGL3 basic (Promega). The recombinant DNA was transformed into *E. coli* and recombinant clones were screened for the presence of cloned promoter insert. Plasmids of the correct size were further analyzed by DNA sequencing using the Big Dye terminator v3.1 cycle sequencing chemistry and ABI capillary sequence.

A quick change Multi Site-directed Mutagenesis kit (Stratagene) was used according to the manufacturer's instructions to create the various *IRF3* promoter constructs (Supplemental Table S3 in Supporting Information S1). 5498 human kidney epithelial cells were cultured in 24-well plates at a density of 2.5×10^5^ cells per well. The cells were transiently transfected with wild type or mutant *IRF3* promoter driven firefly luciferase constructs (pGL3) together with a constitutively expressed internal control construct with Renilla luciferase-thymidine kinase promoter (pRL-TK, Promega) using Fugene HD (Roche) Transfection reagent at 4∶2 ratio. Luciferases were measured using the Dual Luciferase Reporter System Assay (Promega) with a Glomax Integrated Luminometer (Promega). Firefly luciferase data were normalized against transfection efficiency of Renilla luciferase and expressed as a ratio.

### Statistics

Student's *t* test or Wilcoxon's rank-sum test were used for paired comparisons, Mann-Whitney test was applied for unpaired comparisons. P values below 0.05 were considered to indicate statistical significance. Deviations from Hardy-Weinberg equilibrium (HWE) for genotypes at individual loci in patients and controls, as well as differences in genotype and allele distributions between groups, were assessed using the χ^2^ test. Fisher's exact test was used where appropriate.

### List of ID numbers for genes and proteins of mouse and humans

Gene ID number for human TLR4 is 7099, human MyD88 is 4615, human TRIF is 148022, human TRAM is 353376, human TBK1 is 29110, human CREB is 1385, human IRF3 is 3661, mouse Irf3 is 54131, human IFNB is 3456 and mouse Ifnb is 15977.

## Supporting Information

Figure S1 Secondary antibody control of kidney sections containing neutrophils and P fimbriated *E. coli*. Hematoxylin/Eosin staining of abscesses and a corresponding area stained with only secondary goat anti-rat immunoglobulins, conjugated with Alexa fluor-488 and secondary goat anti-rabbit immunoglobulins, conjugated with Alexa fluor-568.
(1.78 MB TIF)

Figure S2 qRT-PCR analysis of knockdown efficiency after siRNA transfection. The knockdown of TLR4, TRAM and MyD88 expression in A549 cells was confirmed by RT-PCR. The mRNA levels were coamplified using GAPDH mRNA as an internal standard. Cells transfected with an irrelevant siRNA (Ctrl) were used as a control. Suppression of TLR4 mRNA and TRAM mRNA was more than 90%, MyD88 mRNA was downregulated by 80%.
(0.09 MB TIF)

Figure S3 Broader field of view of TRAM phosphorylation (TRAM-P) after 30 or 60 minutes of r-ceramide (SMase (1U/ml), C6 ceramide (30µg/ml) or LPS+sCD14 (0.1+1 µg/ml) exposure (primary polyclonal rabbit-TRAM-P antibodies and secondary anti-rabbit-Alexa fluor-568 labelled antibodies).
(1.36 MB TIF)

Figure S4 Identified responders in ceramide/TLR4 induced signalling; a simplified model. P fimbriated *Escherichia coli* use glycosphingolipid receptors to adhere to uroepithelial cells. Binding triggers ceramide release followed by TLR4 and TRAM activation. Downstream signaling involves MAP kinases, CREB, IRF3 nad AP-1 (Jun/Fos). CREB and IRF3 phosphorylation is partly p38 MAPK dependent but not dependent on PKCε and TBK-1.
(1.03 MB TIF)

Figure S5 Genes involved in TLR4 signaling. Panel A shows A549 epithelial cells, stimulated for 1 h with r-ceramide (SMase, 1U/ml) or LPS+sCD14 (10+1µg/ml). Panel B shows A498 epithelial cells stimulated for 3 hours. Relative gene expression was analyzed by RT-PCR-based superarray.
(0.12 MB PDF)

Figure S6 Panel A shows IRF3 staining in J82 human bladder epithelial cells. J82 cells were exposed to r-ceramide (SMase (1U/ml)), LPS (0.1 µg/ml) or LPS+sCD14 (0.1+1 µg/ml) for 90 min and analyzed as described in figure 4A. N = Nuclear staining. Panel B shows Nuclear IRF3 translocation in response to ceramide/TLR4 in A549 cells. IRF3 and NF-κB p65 translocation in 70% confluent A549 cells exposed to r-ceramide (SMase (1U/ml), C6 ceramide (30 µg/ml) or LPS+sCD14 (10+1 µg/ml) for 90 min. N = Nuclear staining.
(1.98 MB TIF)

Figure S7 r-ceramide induced CREB and IRF3 phosphorylation in mouse renal tubular cells (MRTEC) was reduced after treatment with a p38 inhibitor (SB202190). MRTECs were stimulated for 90 min with r-ceramide (SMase, 1U/ml) or LPS+sCD14 (0.1+1µg/ml). Blots of whole cell extracts were stained with phosphospecific rabbit anti-CREB-P- or rabbit anti-IRF3-P- and HRP-conjugated anti-rabbit antibodies. The western blot is a representative of 2 experiments.
(1.98 MB TIF)

Figure S8 Interleukin-8 (IL-8) secretion in A549 cells after treatment with a PKC inhibitor (Bisindolylmaleimide II, 1300 nM) and 24 hours stimulation with r-ceramide (SMase, 2 U/ml), LPS+sCD14 (0.1+1 µg/ml) or PMA (0.01 ng/ml). Means ± SEM of two independent experiments. Med = Medium alone.
(0.15 MB TIF)

Figure S9 Knockdown of TLR4 and TRAM results in abrogation of the ceramide dependent activation of IRF3 phosphorylation while knock down of TBK-1 does not. Western blot analysis after siRNA transfection in A549 cells of TLR4, TRAM or TBK1 siRNA, irrelevant siRNA was used as a control. The knockdown of TLR4, TRAM and TBK1 genes were confirmed by RT-PCR. The knockdown efficiency was more than 90% for TLR4 and TRAM, and 64% for TBK1.
(0.33 MB TIF)

Figure S10 Broader field of view of nuclear translocation of IRF3 and NF-κB in primary human renal tubular epithelial cells after stimulation *E. coli* (N = Nuclear staining, B = Bacteria). The P-fimbriated strain (*E. coli* S1918*pap*) induced higher nuclear IRF3 translocation than non-fimbriated (S1918) and Type 1 fimbriated (S1918*fim*) *E. coli* while NF-κB was translocated in response to all strains, although slightly more in P-fimbriated *E. coli*.
(0.58 MB TIF)

Figure S11 Panel A represents confirmatory experiment of data described in Figure 5A. P-fimbriated *E. coli* (S1918*pap*) induces IRF3 translocation in A498 human kidney epithelial cells more efficiently than cells stimulated with unfimbriated *E. coli* (S1918) and type 1 fimbriated *E. coli* (S1918*fim*) N = Nuclear staining. Panel B - Western blotting showed higher IRF3-P activation in the S1918*pap* infected A498 cells.
(1.02 MB TIF)

Supporting Information S1 Tables S1 to S5.
(0.31 MB PPT)
