# Supplementary figures and images for: Pathogen Specific, IRF3-Dependent Signaling and Innate Resistance to Human Kidney Infection
Source: PLoS Pathog. 2010 Sep 23;6(9):e1001109. doi: 10.1371/journal.ppat.1001109 (PMC2944801; doi:10.1371/journal.ppat.1001109)

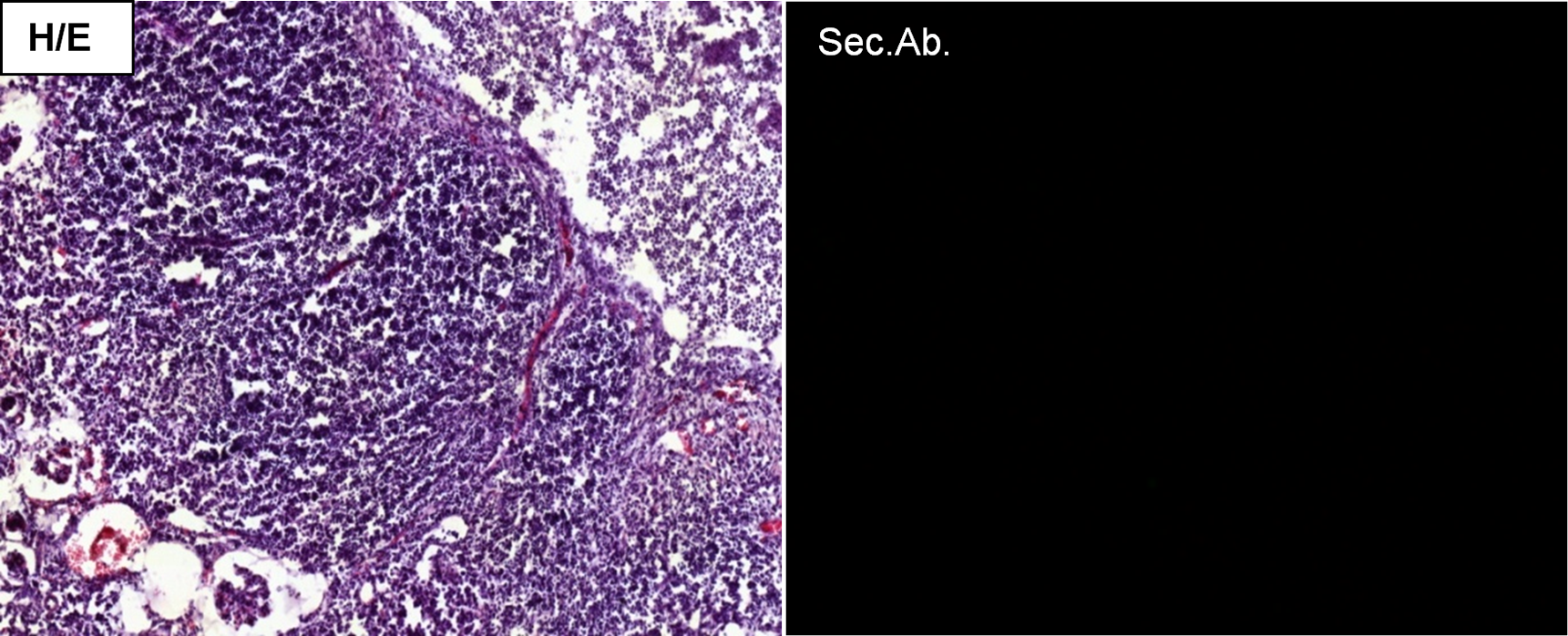

Supplement: Figure S1 — Secondary antibody control of kidney sections containing neutrophils and P fimbriated E. coli. Hematoxylin/Eosin staining of abscesses and a corresponding area stained with only secondary goat anti-rat immunoglobulins, conjugated with Alexa fluor-488 and secondary goat anti-rabbit immunoglobulins, conjugated with Alexa fluor-568. (1.78 MB TIF) [file ppat.1001109.s001.tif]

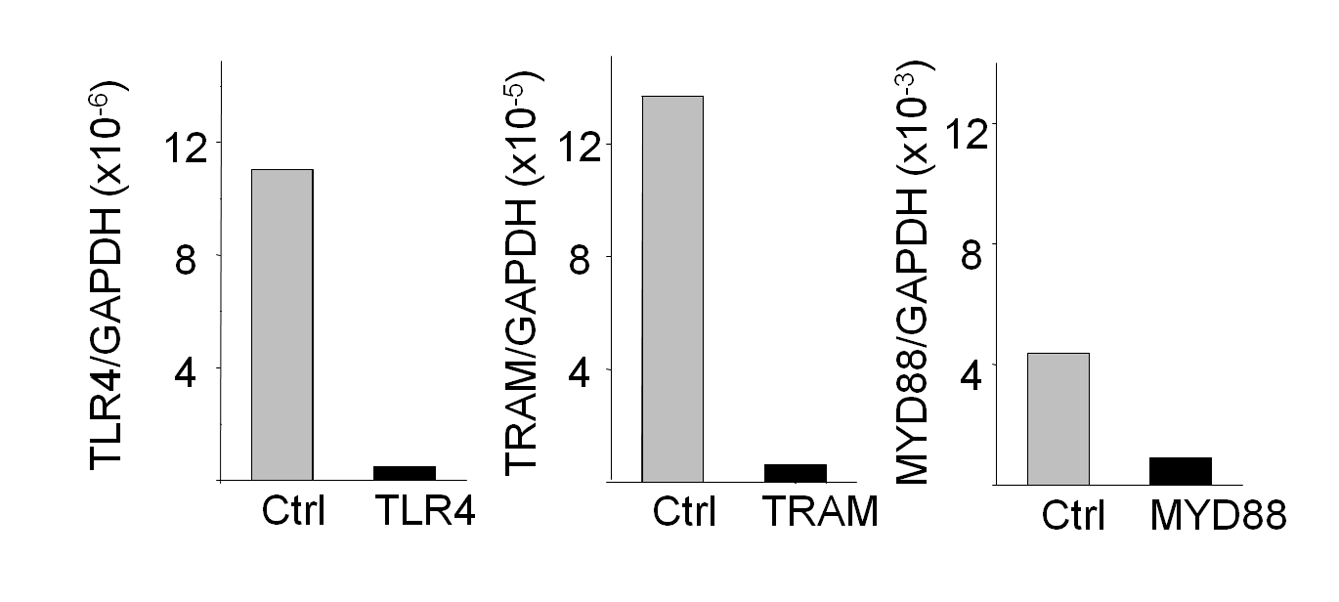

Supplement: Figure S2 — qRT-PCR analysis of knockdown efficiency after siRNA transfection. The knockdown of TLR4, TRAM and MyD88 expression in A549 cells was confirmed by RT-PCR. The mRNA levels were coamplified using GAPDH mRNA as an internal standard. Cells transfected with an irrelevant siRNA (Ctrl) were used as a control. Suppression of TLR4 mRNA and TRAM mRNA was more than 90%, MyD88 mRNA was downregulated by 80%. (0.09 MB TIF) [file ppat.1001109.s002.tif]

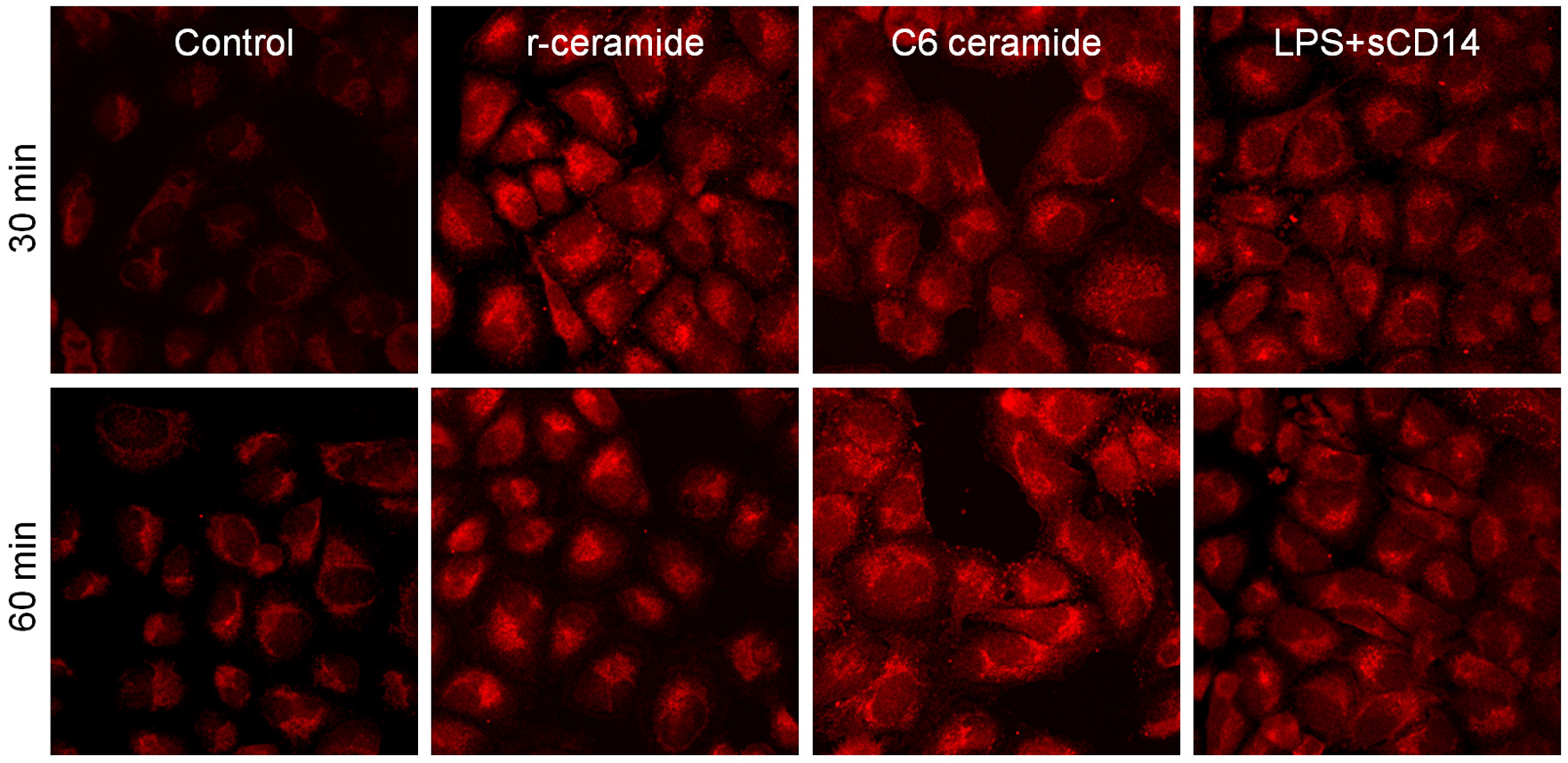

Supplement: Figure S3 — Broader field of view of TRAM phosphorylation (TRAM-P) after 30 or 60 minutes of r-ceramide (SMase (1U/ml), C6 ceramide (30µg/ml) or LPS+sCD14 (0.1+1 µg/ml) exposure (primary polyclonal rabbit-TRAM-P antibodies and secondary anti-rabbit-Alexa fluor-568 labelled antibodies). (1.36 MB TIF) [file ppat.1001109.s003.tif]

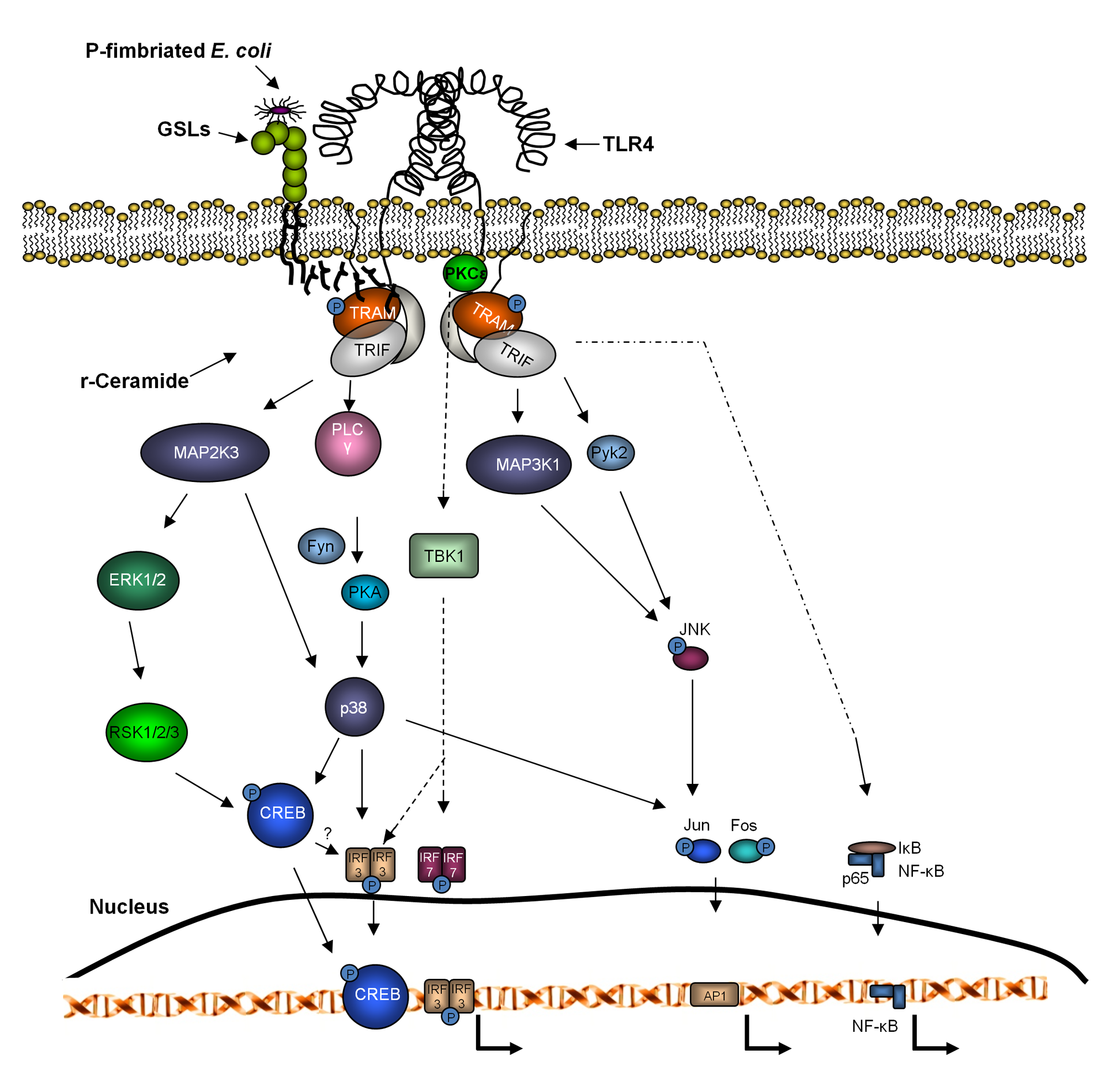

Supplement: Figure S4 — Identified responders in ceramide/TLR4 induced signalling; a simplified model. P fimbriated Escherichia coli use glycosphingolipid receptors to adhere to uroepithelial cells. Binding triggers ceramide release followed by TLR4 and TRAM activation. Downstream signaling involves MAP kinases, CREB, IRF3 nad AP-1 (Jun/Fos). CREB and IRF3 phosphorylation is partly p38 MAPK dependent but not dependent on PKCε and TBK-1. (1.03 MB TIF) [file ppat.1001109.s004.tif]

A

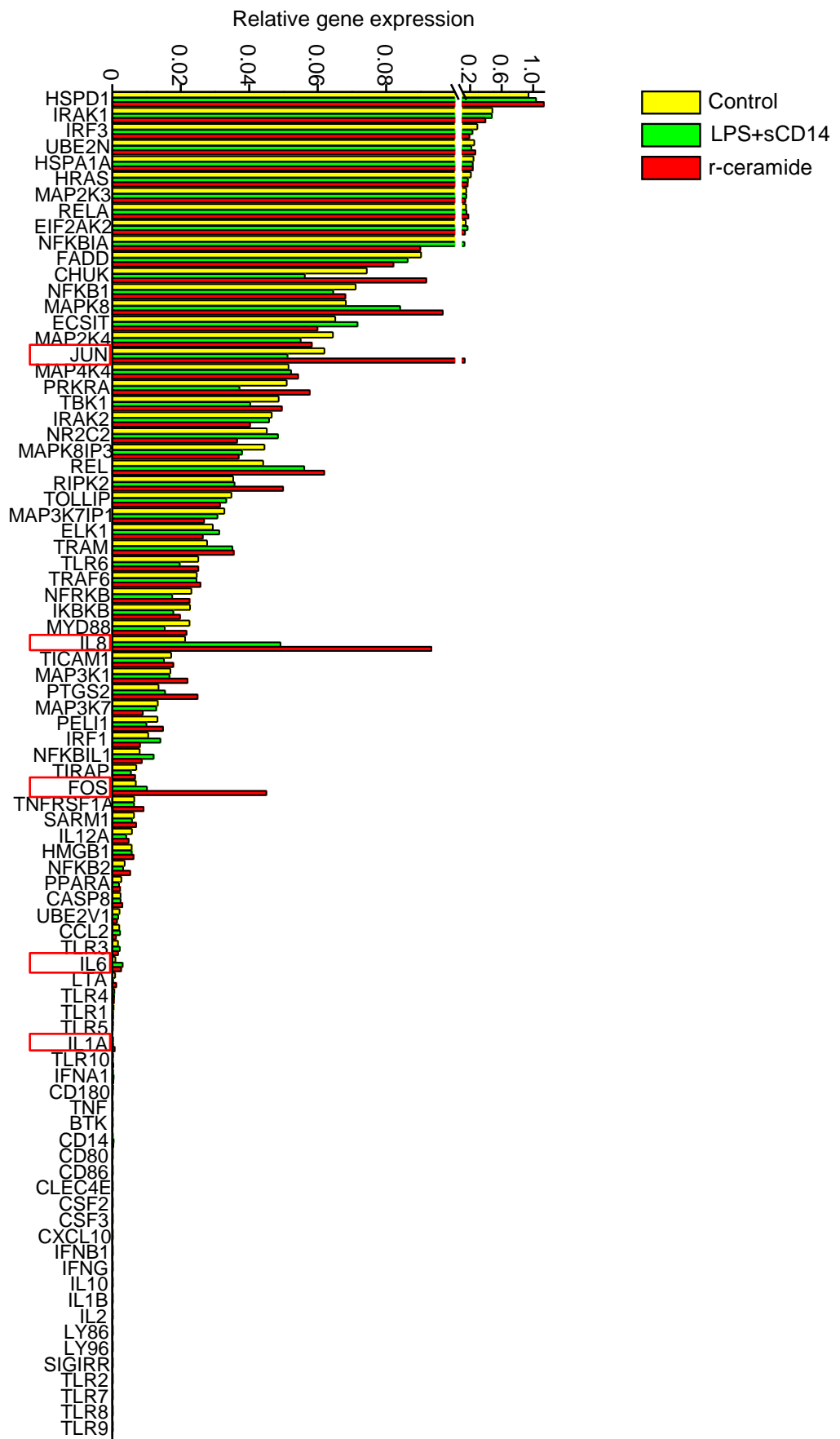

**B**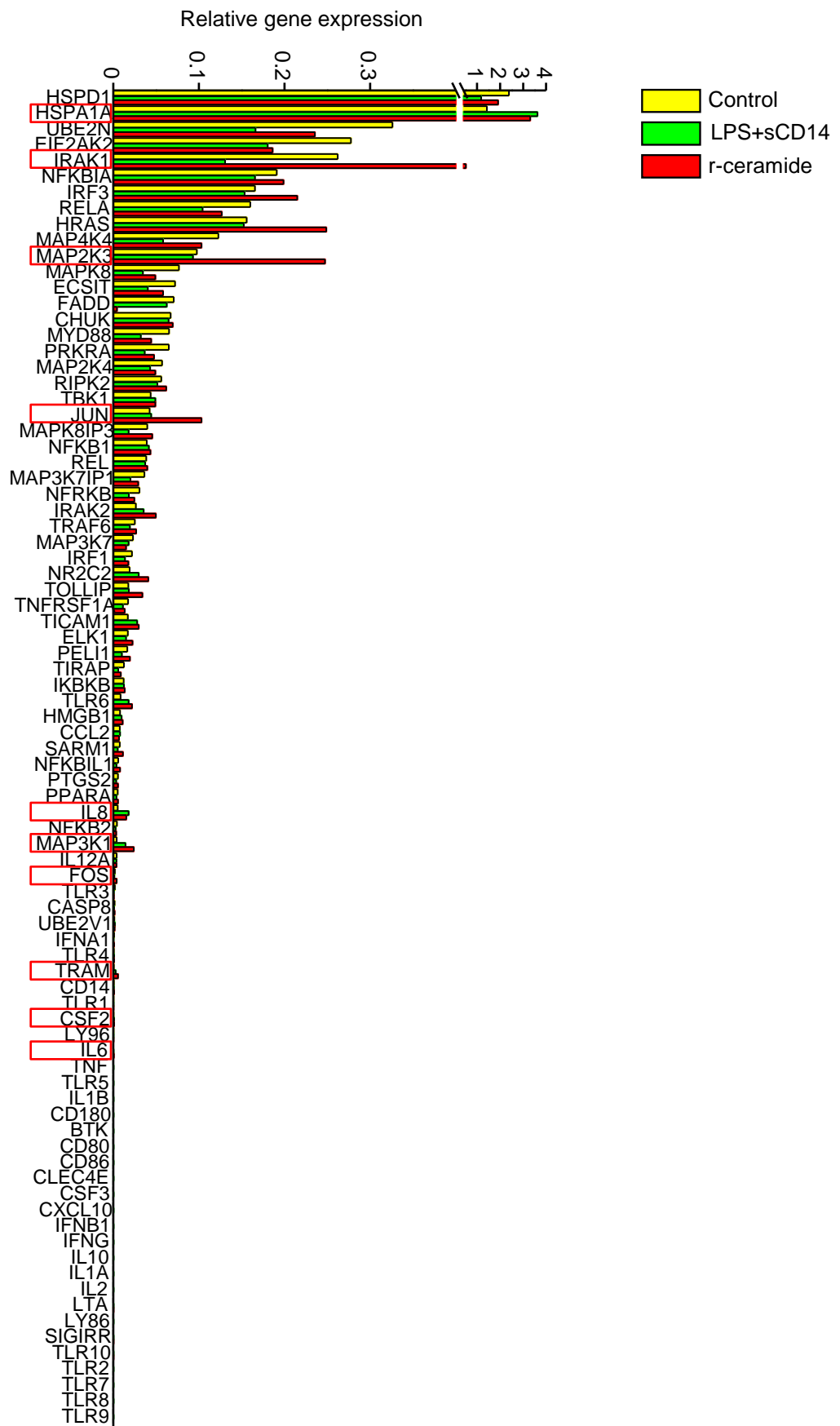

Supplement: Figure S5 — Genes involved in TLR4 signaling. Panel A shows A549 epithelial cells, stimulated for 1 h with r-ceramide (SMase, 1U/ml) or LPS+sCD14 (10+1µg/ml). Panel B shows A498 epithelial cells stimulated for 3 hours. Relative gene expression was analyzed by RT-PCR-based superarray. (0.12 MB PDF) [file ppat.1001109.s005.pdf]

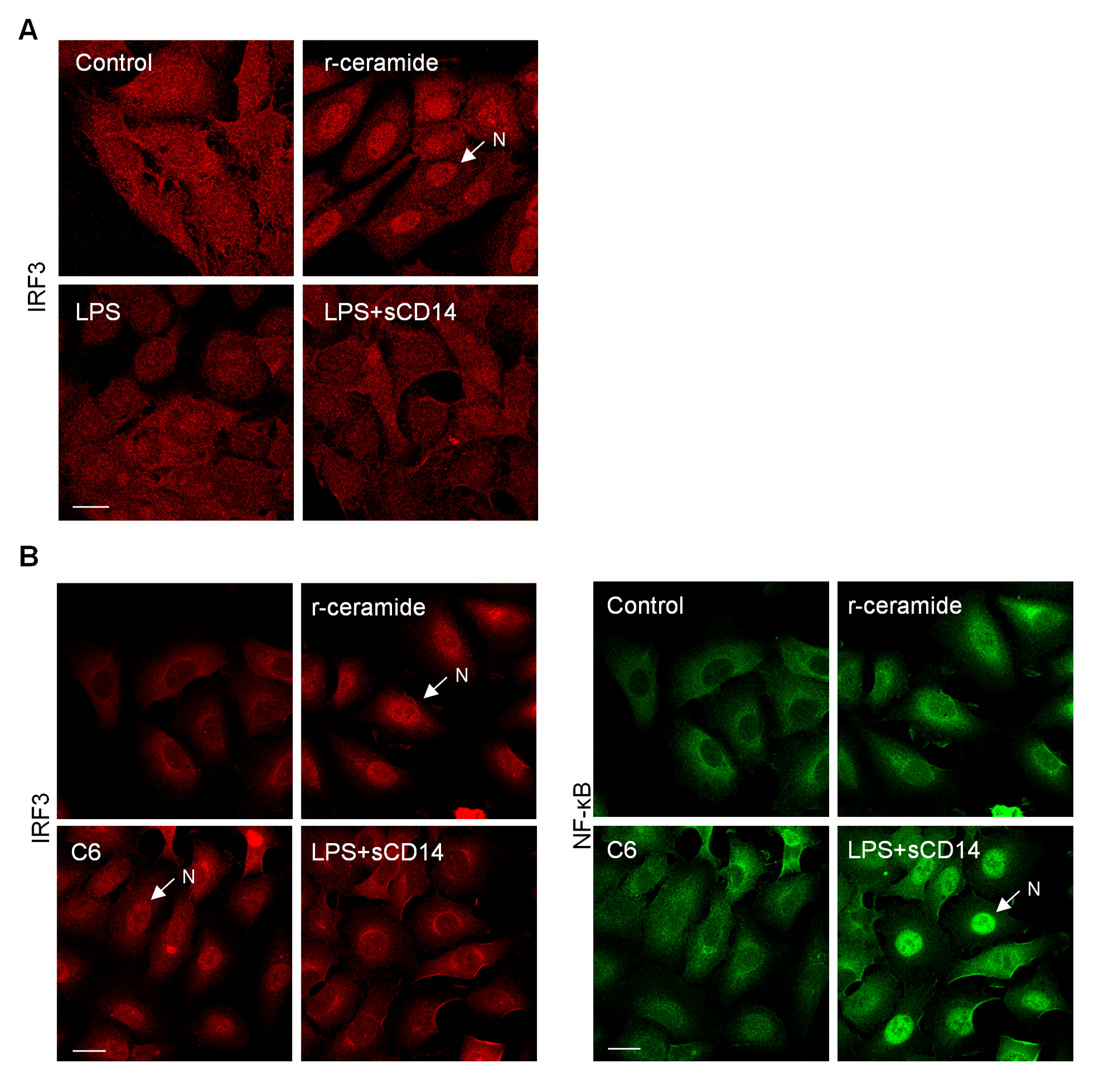

Supplement: Figure S6 — Panel A shows IRF3 staining in J82 human bladder epithelial cells. J82 cells were exposed to r-ceramide (SMase (1U/ml)), LPS (0.1 µg/ml) or LPS+sCD14 (0.1+1 µg/ml) for 90 min and analyzed as described in figure 4A. N = Nuclear staining. Panel B shows Nuclear IRF3 translocation in response to ceramide/TLR4 in A549 cells. IRF3 and NF-κB p65 translocation in 70% confluent A549 cells exposed to r-ceramide (SMase (1U/ml), C6 ceramide (30 µg/ml) or LPS+sCD14 (10+1 µg/ml) for 90 min. N = Nuclear staining. (1.98 MB TIF) [file ppat.1001109.s006.tif]

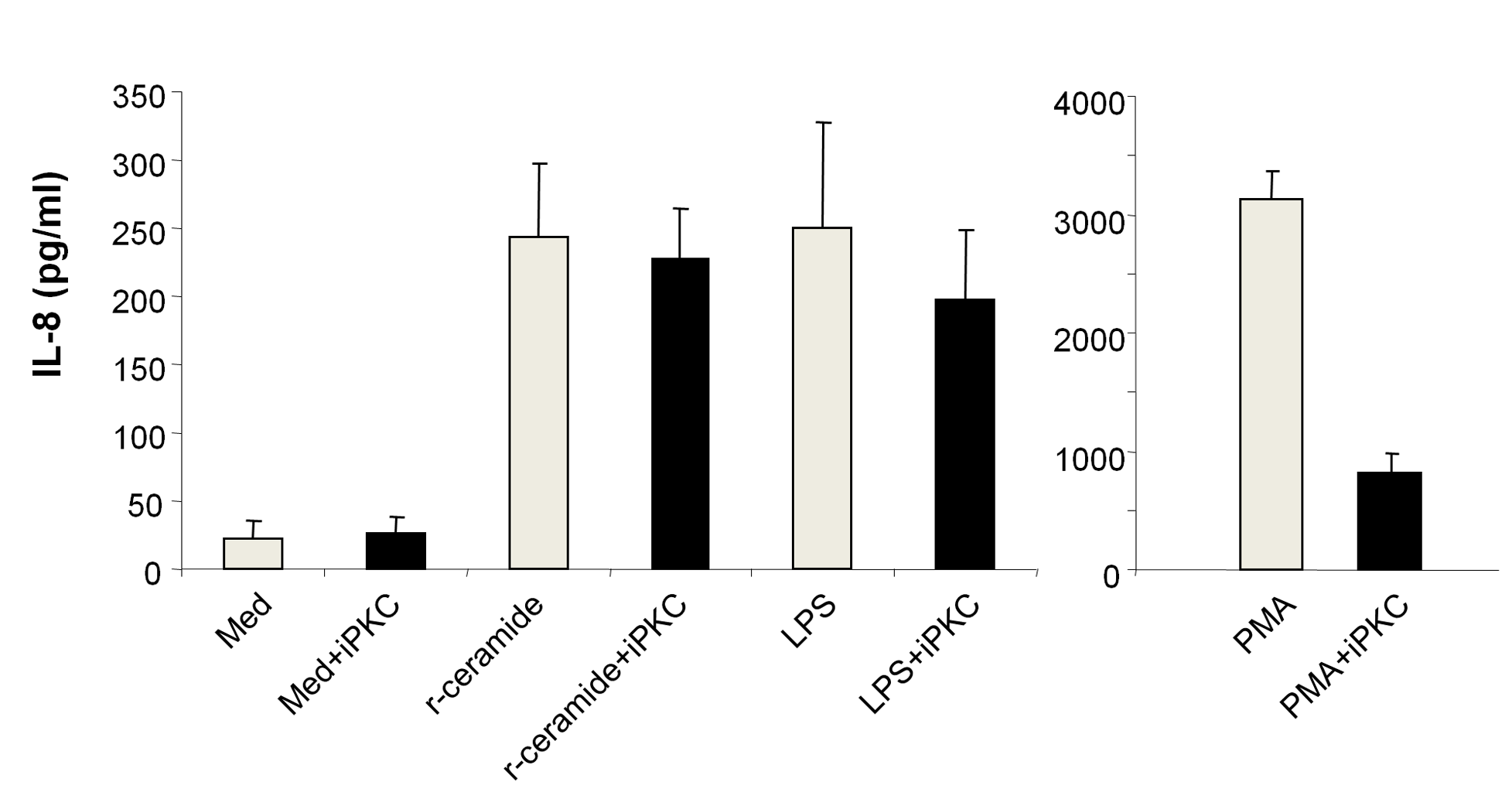

Supplement: Figure S8 — Interleukin-8 (IL-8) secretion in A549 cells after treatment with a PKC inhibitor (Bisindolylmaleimide II, 1300 nM) and 24 hours stimulation with r-ceramide (SMase, 2 U/ml), LPS+sCD14 (0.1+1 µg/ml) or PMA (0.01 ng/ml). Means ± SEM of two independent experiments. Med = Medium alone. (0.15 MB TIF) [file ppat.1001109.s008.tif]

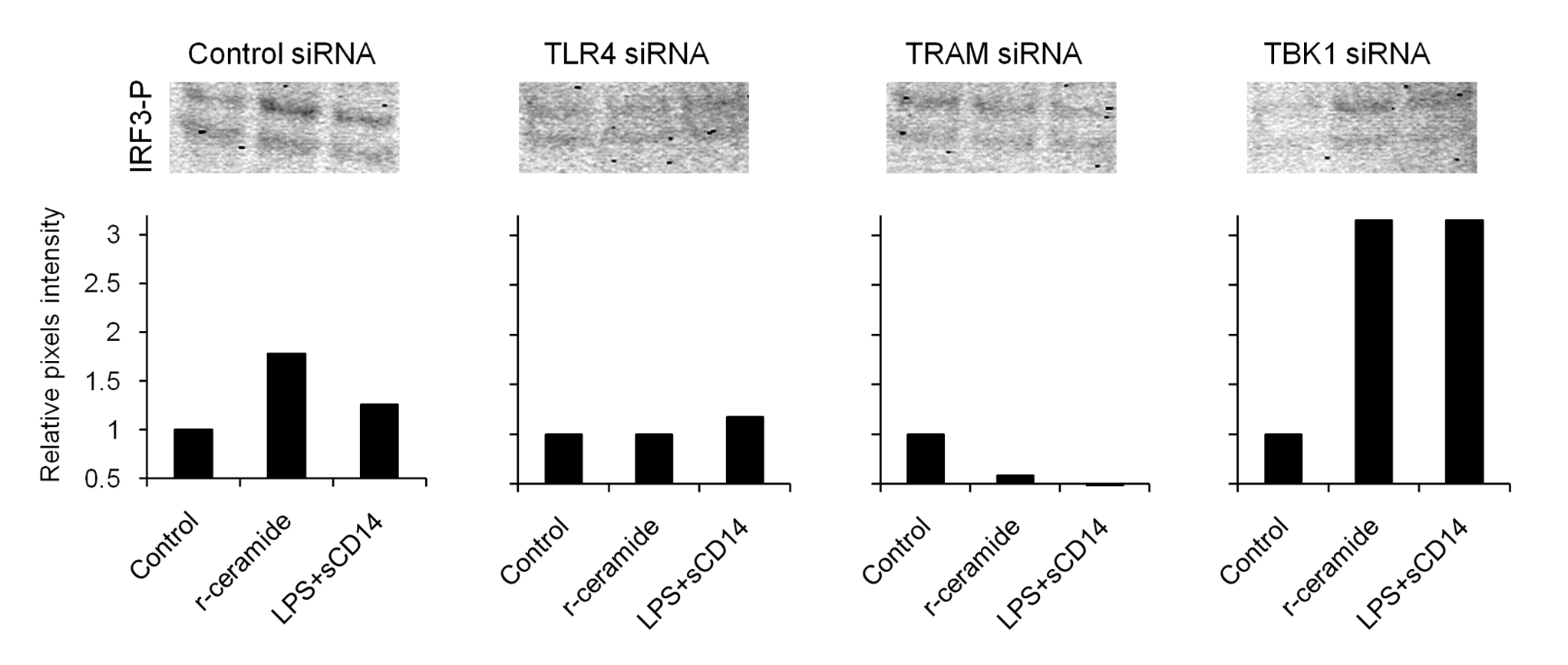

Supplement: Figure S9 — Knockdown of TLR4 and TRAM results in abrogation of the ceramide dependent activation of IRF3 phosphorylation while knock down of TBK-1 does not. Western blot analysis after siRNA transfection in A549 cells of TLR4, TRAM or TBK1 siRNA, irrelevant siRNA was used as a control. The knockdown of TLR4, TRAM and TBK1 genes were confirmed by RT-PCR. The knockdown efficiency was more than 90% for TLR4 and TRAM, and 64% for TBK1. (0.33 MB TIF) [file ppat.1001109.s009.tif]

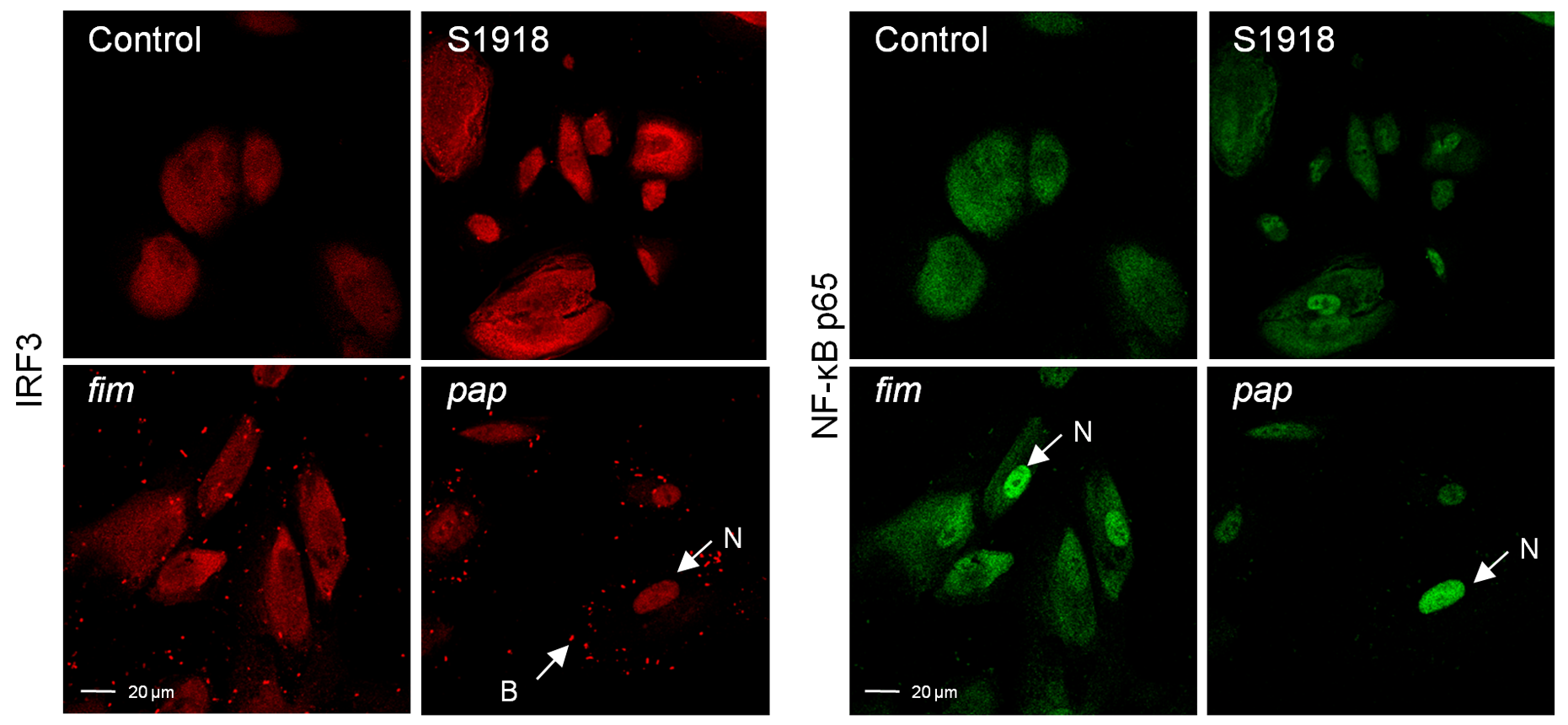

Supplement: Figure S10 — Broader field of view of nuclear translocation of IRF3 and NF-κB in primary human renal tubular epithelial cells after stimulation E. coli (N = Nuclear staining, B = Bacteria). The P-fimbriated strain (E. coli S1918pap) induced higher nuclear IRF3 translocation than non-fimbriated (S1918) and Type 1 fimbriated (S1918fim) E. coli while NF-κB was translocated in response to all strains, although slightly more in P-fimbriated E. coli. (0.58 MB TIF) [file ppat.1001109.s010.tif]

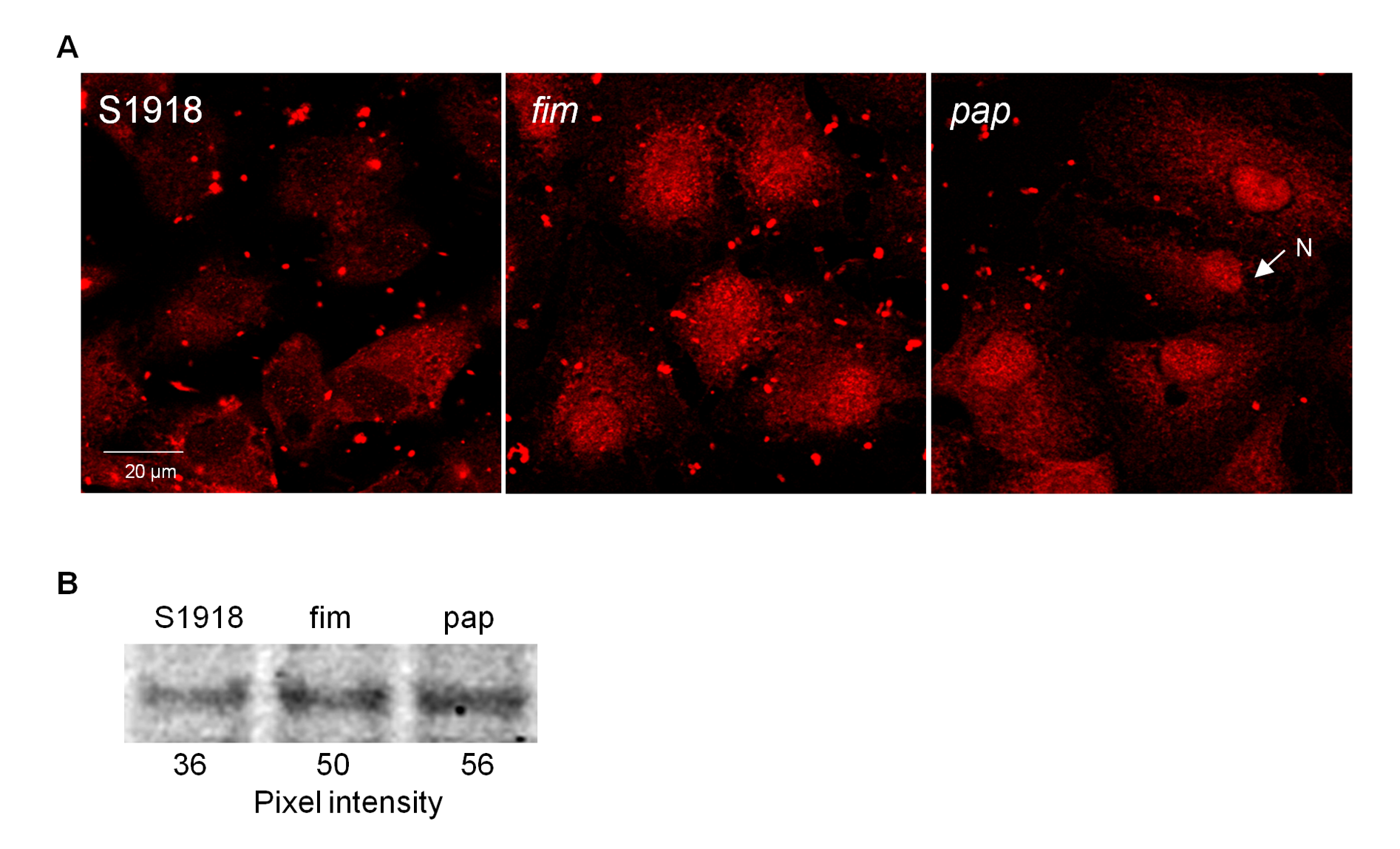

Supplement: Figure S11 — Panel A represents confirmatory experiment of data described in Figure 5A. P-fimbriated E. coli (S1918pap) induces IRF3 translocation in A498 human kidney epithelial cells more efficiently than cells stimulated with unfimbriated E. coli (S1918) and type 1 fimbriated E. coli (S1918fim) N = Nuclear staining. Panel B - Western blotting showed higher IRF3-P activation in the S1918pap infected A498 cells. (1.02 MB TIF) [file ppat.1001109.s011.tif]
