# Supplementary material for: Pathogen Specific, IRF3-Dependent Signaling and Innate Resistance to Human Kidney Infection
Source: PLoS Pathog. 2010 Sep 23;6(9):e1001109. doi: 10.1371/journal.ppat.1001109 (PMC2944801; doi:10.1371/journal.ppat.1001109)
Supplement: Supporting Information S1 — Tables S1 to S5. (0.31 MB PPT) [file ppat.1001109.s012.ppt]

## Slide 1
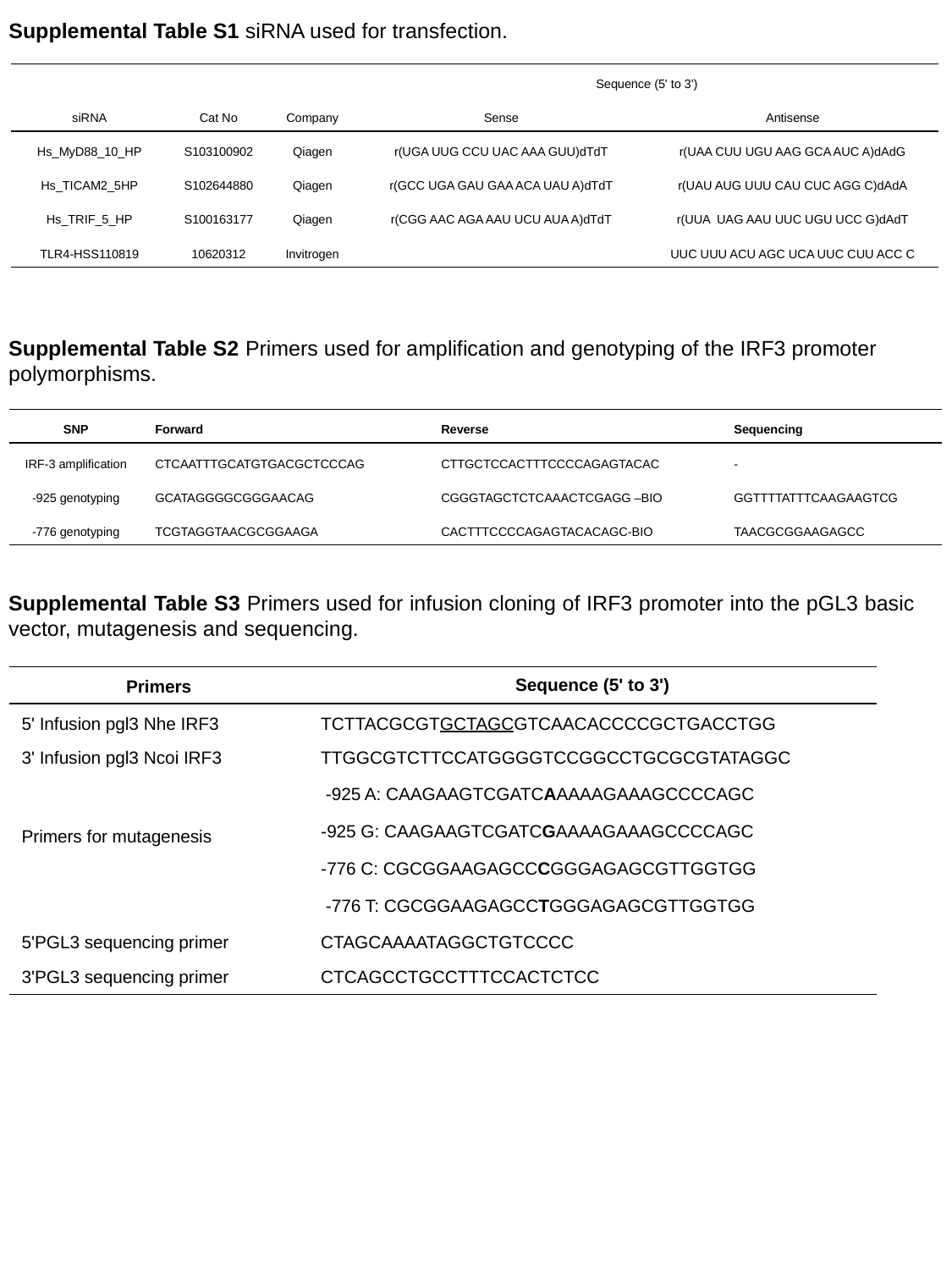

Supplemental Table S1 siRNA used for transfection.
| | | | Sequence (5' to 3') | |
| --- | --- | --- | --- | --- |
| siRNA | Cat No | Company | Sense | Antisense |
| Hs\_MyD88\_10\_HP | S103100902 | Qiagen | r(UGA UUG CCU UAC AAA GUU)dTdT | r(UAA CUU UGU AAG GCA AUC A)dAdG |
| Hs\_TICAM2\_5HP | S102644880 | Qiagen | r(GCC UGA GAU GAA ACA UAU A)dTdT | r(UAU AUG UUU CAU CUC AGG C)dAdA |
| Hs\_TRIF\_5\_HP | S100163177 | Qiagen | r(CGG AAC AGA AAU UCU AUA A)dTdT | r(UUA UAG AAU UUC UGU UCC G)dAdT |
| TLR4-HSS110819 | 10620312 | Invitrogen | | UUC UUU ACU AGC UCA UUC CUU ACC C |
Supplemental Table S2 Primers used for amplification and genotyping of the IRF3 promoter polymorphisms.
| SNP | Forward | Reverse | Sequencing |
| --- | --- | --- | --- |
| IRF-3 amplification | CTCAATTTGCATGTGACGCTCCCAG | CTTGCTCCACTTTCCCCAGAGTACAC | - |
| -925 genotyping | GCATAGGGGCGGGAACAG | CGGGTAGCTCTCAAACTCGAGG –BIO | GGTTTTATTTCAAGAAGTCG |
| -776 genotyping | TCGTAGGTAACGCGGAAGA | CACTTTCCCCAGAGTACACAGC-BIO | TAACGCGGAAGAGCC |
Supplemental Table S3 Primers used for infusion cloning of IRF3 promoter into the pGL3 basic vector, mutagenesis and sequencing.
| Primers | Sequence (5' to 3') |
| --- | --- |
| 5' Infusion pgl3 Nhe IRF3 | TCTTACGCGTGCTAGCGTCAACACCCCGCTGACCTGG |
| 3' Infusion pgl3 Ncoi IRF3 | TTGGCGTCTTCCATGGGGTCCGGCCTGCGCGTATAGGC |
| Primers for mutagenesis | -925 A: CAAGAAGTCGATCAAAAAGAAAGCCCCAGC |
| | -925 G: CAAGAAGTCGATCGAAAAGAAAGCCCCAGC |
| | -776 C: CGCGGAAGAGCCCGGGAGAGCGTTGGTGG |
| | -776 T: CGCGGAAGAGCCTGGGAGAGCGTTGGTGG |
| 5'PGL3 sequencing primer | CTAGCAAAATAGGCTGTCCCC |
| 3'PGL3 sequencing primer | CTCAGCCTGCCTTTCCACTCTCC |

## Slide 2
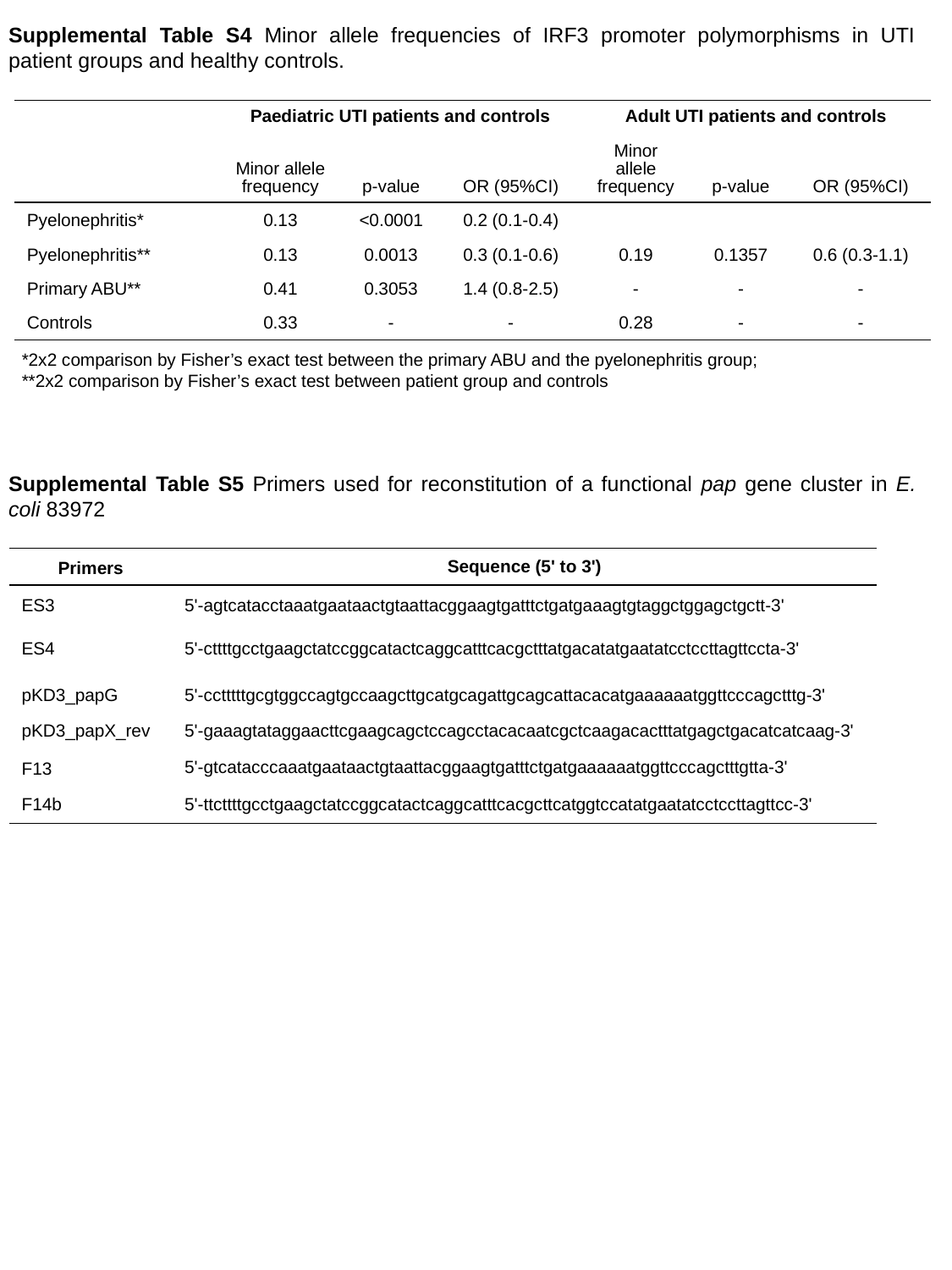

Supplemental Table S4 Minor allele frequencies of IRF3 promoter polymorphisms in UTI patient groups and healthy controls.
| | Paediatric UTI patients and controls | | | Adult UTI patients and controls | | |
| --- | --- | --- | --- | --- | --- | --- |
| | Minor allele frequency | p-value | OR (95%CI) | Minor allele frequency | p-value | OR (95%CI) |
| Pyelonephritis\* | 0.13 | <0.0001 | 0.2 (0.1-0.4) | | | |
| Pyelonephritis\*\* | 0.13 | 0.0013 | 0.3 (0.1-0.6) | 0.19 | 0.1357 | 0.6 (0.3-1.1) |
| Primary ABU\*\* | 0.41 | 0.3053 | 1.4 (0.8-2.5) | - | - | - |
| Controls | 0.33 | - | - | 0.28 | - | - |
*2x2 comparison by Fisher’s exact test between the primary ABU and the pyelonephritis group;
**2x2 comparison by Fisher’s exact test between patient group and controls
Supplemental Table S5 Primers used for reconstitution of a functional pap gene cluster in E. coli 83972
| Primers | Sequence (5' to 3') |
| --- | --- |
| ES3 | 5'-agtcatacctaaatgaataactgtaattacggaagtgatttctgatgaaagtgtaggctggagctgctt-3' |
| ES4 | 5'-cttttgcctgaagctatccggcatactcaggcatttcacgctttatgacatatgaatatcctccttagttccta-3' |
| pKD3\_papG | 5'-cctttttgcgtggccagtgccaagcttgcatgcagattgcagcattacacatgaaaaaatggttcccagctttg-3' |
| pKD3\_papX\_rev | 5'-gaaagtataggaacttcgaagcagctccagcctacacaatcgctcaagacactttatgagctgacatcatcaag-3' |
| F13 | 5'-gtcatacccaaatgaataactgtaattacggaagtgatttctgatgaaaaaatggttcccagctttgtta-3' |
| F14b | 5'-ttcttttgcctgaagctatccggcatactcaggcatttcacgcttcatggtccatatgaatatcctccttagttcc-3' |
